# Polarized actin and VE-cadherin dynamics regulate junctional remodelling and cell migration during sprouting angiogenesis

**DOI:** 10.1038/s41467-017-02373-8

**Published:** 2017-12-20

**Authors:** Jiahui Cao, Manuel Ehling, Sigrid März, Jochen Seebach, Katsiaryna Tarbashevich, Tomas Sixta, Mara E. Pitulescu, Ann-Cathrin Werner, Boris Flach, Eloi Montanez, Erez Raz, Ralf H. Adams, Hans Schnittler

**Affiliations:** 1 Institute of Anatomy and Vascular Biology, Westfälische Wilhelms University of Münster, Faculty of Medicine, D-48149 Münster, Germany; 2 Max Planck Institute for Molecular Biomedicine and Westfälische Wilhelms University of Münster, Faculty of Medicine, D-48149 Münster, Germany; 3 Institute of Cell Biology, Center for Molecular Biology of Inflammation, D-48149 Münster, Germany; 4 Department of Cybernetics, Czech Technical University, 16627 Prague 6, Czech Republic; 5 Walter-Brendel-Centre of Experimental Medicine, University Hospital, LMU Munich, D-81377 Munich, Germany

## Abstract

VEGFR-2/Notch signalling regulates angiogenesis in part by driving the remodelling of endothelial cell junctions and by inducing cell migration. Here, we show that VEGF-induced polarized cell elongation increases cell perimeter and decreases the relative VE-cadherin concentration at junctions, triggering polarized formation of actin-driven junction-associated intermittent lamellipodia (JAIL) under control of the WASP/WAVE/ARP2/3 complex. JAIL allow formation of new VE-cadherin adhesion sites that are critical for cell migration and monolayer integrity. Whereas at the leading edge of the cell, large JAIL drive cell migration with supportive contraction, lateral junctions show small JAIL that allow relative cell movement. VEGFR-2 activation initiates cell elongation through dephosphorylation of junctional myosin light chain II, which leads to a local loss of tension to induce JAIL-mediated junctional remodelling. These events require both microtubules and polarized Rac activity. Together, we propose a model where polarized JAIL formation drives directed cell migration and junctional remodelling during sprouting angiogenesis.

## Abstract (web summary)

The formation of new blood vessels requires both polarized cell migration and coordinated control of endothelial cell contacts. Here, Cao and colleagues describe at the sub-cellular level the cytoskeletal and cell junction dynamics regulating these processes upon VEGF-induced cell elongation.

## Introduction

The formation of new blood vessels from a pre-existing vascular network, angiogenesis, involves the participation of endothelial cells (ECs), stromal cells (like fibroblasts), pericytes and the control of extracellular matrix turnover, all of which are coordinated by many angiogenic and angiostatic factors. Angiogenesis is frequently initiated by a gradient of vascular endothelial growth factor (VEGF) that leads to the induction of endothelial-derived tip cells, which extend filopodia and lead sprouts during their extension into VEGF-expressing tissues^1^. Proliferating stalk cells allow vessel growth to follow migrating tip cells^1^. Tip and stalk cells are thought to interconvert dynamically, leading to changes in the leading cell position^2^. Cell migration and positional changes by angiogenic ECs require closely coordinated interactions of cell junctions that critically depend on the dynamics of vascular endothelial cadherin (VE-cadherin)^3–5^. In particular, a moderate decrease in VE-cadherin expression enhances angiogenic sprouting in vitro^6^ and in the murine retina in vivo, as demonstrated by inducible inactivation of the cadherin 5 (*Cdh5)* gene encoding VE-cadherin^3^. VE-cadherin is also essential for proper vessel growth and lumen formation, and, accordingly, lumen development is disrupted in Cdh5 knockout embryos^7^. Moreover, VE-cadherin was shown to suppress VEGFR2-Rac1-dependent vessel sprouting^6^. These data indicate that an appropriate VE-cadherin concentration at cell junctions is critical for proper angiogenesis.

Despite these insights, increasing vessel size and lumen formation by stalk cells increases the number of cell contacts by which a particular cell connects to several other cells, including the tip cell/stalk cell interaction, e.g., during interconversion. This important aspect becomes even more important, as stalk cells not only move forward but can also migrate backwards in an existing vessel-like structure^2^, a fact that requires, apart from individually regulated junctions, a reversal of cell polarity. A structure that gave some insight into this aspect was described in a recent cell culture study, demonstrating that local reduction of VE-cadherin at particular endothelial junction sites initiates the formation of actin-driven plasma membrane protrusions. Due to their transient, spatiotemporally restricted and highly dynamic features, these structures were termed junction-associated intermittent lamellipodia (JAIL)^8^. JAIL, in turn, induce new VE-cadherin adhesion sites and thereby drive VE-cadherin and actin dynamics interdependently, resulting in a coupling of VE-cadherin and actin dynamics in migrating ECs. Furthermore, JAIL are controlled by WAVE/WASP/ARP2/3 complex and ensure endothelial integrity^8^, which is consistent with WAVE/WASP-mediated docking structure formation during lymphocyte transmigration and maintained barrier function^9^. In addition, a recent work demonstrated differential VE-cadherin patterning characterised by “junctional cortex” protrusions, which can be induced by the pro-angiogenic factor VEGF or the inhibition of Notch signalling^4^. On the basis of the results presented here, we were able to develop a model of cell junction dynamics for VEGFR2/Notch-mediated polar cell migration during angiogenesis that includes contraction/relaxation mechanisms and interdependent dynamics of microtubules, VE-cadherin and actin.

## Results

### Cell elongation reduces relative VE-cadherin concentration

**Fig. 1:**
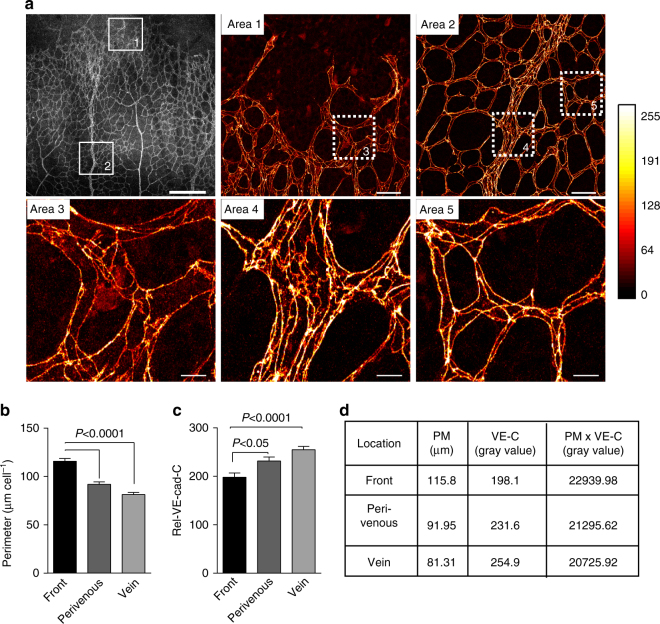
Migrating front ECs in developing retinas demonstrate an increased perimeter and a reduced Rel-VEcad-C. **a** Representative heat-map images of VE-cadherin-labelled ECs in front and centre areas of whole-mounted P6 mouse retinas. Area 1 and Area 2 (scale bar: 40 µm), depicted as indicated, display higher magnifications of the angiogenic front and centre vein area, respectively. Area 3 to Area 5 (scale bar: 20 µm) correspond to the dotted white boxes in Area 1 and 2, respectively. **b**, **c** Quantification of cell perimeter and Rel-VEcad-C in angiogenic front, perivenous plexus in the centre, and vein ECs. Three independent experiments with n cells (*n* = 88, 101, and 106) were used for cell perimeter quantification and (*n* = 54, 58, and 75) for Rel-VEcad-C. **d** Estimation of the total VE-cadherin amount in ECs was performed by multiplying the mean cell perimeter by the mean Rel-VEcad-C. One-way ANOVA was used to analyse the significance difference. Error bars represent ± SEM. PM perimeter, VE-C Rel-VEcad-C

VE-cadherin and actin dynamics at EC junctions are upregulated in cells with increased cell junction length via JAIL formation, which is activated by a reduced relative VE-cadherin concentration (Rel-VEcad-C) at extended cell junctions, although the total amount of VE-cadherin remains unchanged as demonstrated in human umbilical vein endothelial cell (HUVEC) cultures^8^. Suggesting that a similar mechanism plays a role during angiogenesis, we found in VE-cadherin-labelled P6 mice retinas an increased EC junction length at the angiogenic front compared with those in the vein or in the perivenous capillaries (Fig. 1a, b). Cell elongation caused a decrease in Rel-VEcad-C of 22% compared with in EC of the central veins and of 14.5% compared with the mature perivenous EC (capillary plexus) (Fig. 1a, c). The product of the mean Rel-VEcad-C and the corresponding mean cell perimeter confirmed that the total VE-cadherin content per cell was largely constant (Fig. 1d). Thus, we hypothesised that the decrease in Rel-VEcad-C could promote cell junction dynamics and cell motility in angiogenesis in vivo by JAIL formation.

### JAIL drive junction dynamics in sheet migration

**Fig. 2:**
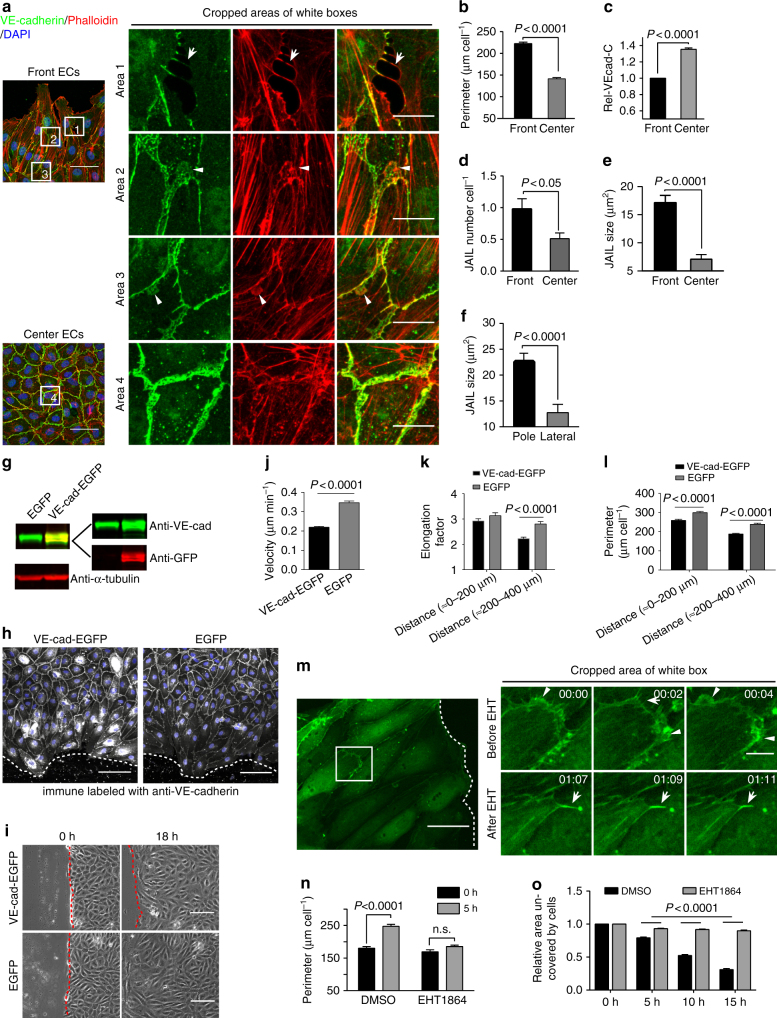
JAIL formation and cell migration are blocked by Rac inhibition and VE-cadherin overexpression in scratch assay. **a**–**f** HUVECs were scratched, allowed to grow for 5 h, and labelled for VE-cadherin and Phalloidin-TRITC. **a** Overviews of the migrating front (left upper) and centre area (left lower). (Area 1) interrupted VE-cadherin that co-localise with filopodia-like actin filaments (arrows). (Area 2) Large JAIL (arrowheads) at cell pole. (Area 3) small JAIL (arrowheads) at lateral junctions. (Area 4) Polygonal cells in the centre area. Scale bars represent 60 µm and 15 µm in the overview and cropped images respectively. **b**, **c** Comparison of cell perimeter and Rel-VEcad-C in front and centre ECs. Quantification of (**d**) JAIL number and (**e**) JAIL size in front (*n* = 115 cells) and centre cells (*n* = 186 cells). **f** JAIL size at cell poles and lateral junctions in front ECs (*n* = 115 cells). *P* value was determined by unpaired student’s *t* test. **g** Western blot demonstrates VE-cadherin-EGFP overexpression by a factor of about 4 using VE-cadherin (green) and EGFP (red) antibodies. **h** VE-cadherin was immune labelled in VE-cadherin-EGFP-overexpressing and EGFP-expressing cells; nuclei were stained blue with DAPI. Scale bar: 100 µm. **i** Representative images of VE-cadherin-EGFP-overexpressing and EGFP-expressing cells in a scratch assay. Red dotted lines indicate wound margins. Scale bar: 150 µm. **j** Quantification of migration velocity 18.5 h after the scratch (*n* = 83 and 85 cells, respectively, unpaired student’s *t* test). **k**, **l** Quantification of elongation factor and cell perimeter using CBT based on immunofluorescence images [*n* = 176 and 205 cells for VE-cadherin-EGFP-transfected HUVECs, and *n* = 139 and 165 cells for EGFP-expressing HUVECs at distance (≈ 0–200) and (≈ 200–400) in the scratch area, two-way ANOVA]. **m** Time-lapse series of HUVECs expressing LifeAct-EGFP after scratching. (Left) Overview image (scale bar: 50 µm) indicates the scratch front (dotted line) and the white box represents the cropped area. JAIL formation (arrowheads) in the same cells was blocked by EHT1864, while filopodia still developed (arrows). Time scale: hh:mm, scale bar: 10 µm. Live-cell imaging-based analyses of (**n**) cell perimeter (front to 200 µm, *n* = 50 cells) and (**o**) wound closure (*n* = 3) (two-way ANOVA). Representative results from three independent experiments were shown. Error bars represent ± SEM. VE-cad-EGFP VE-cadherin-EGFP

After scratching a wound in confluent HUVEC cultures, classical lamellipodia appeared on the front cells (leaders), followed by directed cell migration as described previously^10^. Most followers responded with shape change, mainly by cell elongation and directed cell migration. A number of cells, however, transiently changed direction and moved backwards or appeared to be stationary. The overall monolayer integrity was conserved throughout the wound closure (Supplementary Movie 1). Immune-stained endogenous VE-cadherin and actin in scratched cultures (5 h after scratch) displayed disassembled junction-associated actin filaments (JAAF) and stress fibre formation in EC at the front area, whereas centred cells largely retained JAAF (Fig. 2a). Small intercellular gaps appeared occasionally, preferentially between leader cells, which were quickly closed by JAIL (Fig. 2a), a process that was reported in previous studies^8,11,12^. The increased cell perimeter (up to 156%) of front cells (Fig. 2b) was accompanied by a decrease in Rel-VEcad-C down to 73.8% (200 µm from the wound edge) (Fig. 2c). Furthermore, elongated cells displayed an increase in JAIL size and number (Fig. 2d, e), with large JAIL appearing at the cell poles while small JAIL were characteristic for the lateral junctions (Fig. 2f).

To closely follow JAIL-mediated cell junction dynamics, VE-cadherin-EGFP or VE-cadherin-mCherry and EGFP-p20, a fluorescence-tagged subdomain of the ARP2/3 complex^13^, were co-expressed. Live-cell imaging of migrating cells confirmed constitutive VE-cadherin dynamics with formation of linear and interrupted VE-cadherin intermediates (Supplementary Movie 2). Importantly, VE-cadherin plaques appeared transiently at cell junctions (Supplementary Movie 2). These plaques developed after actin-driven and ARP2/3 complex-controlled protrusions (JAIL) that formed at sites of reduced Rel-VEcad-C (e.g., as an interrupted pattern). The interrupted VE-cadherin pattern at the cell poles of elongated cells accompanied large JAIL, whereas the linear VE-cadherin pattern at lateral junctions were associated with small JAIL only (Supplementary Movie 3). Since actin-driven JAIL overlap the corresponding plasma membrane of the neighbouring cell, forming VE-cadherin adhesion that appeared as VE-cadherin plaques (Supplementary Movie 3). The dissociation of branched actin filaments and the ARP2/3 complex (EGFP-p20) correlated with termination of JAIL formation. Subsequently, the VE-cadherin plaques clustered during JAIL retraction and integrated into the cell contacts (Supplementary Movie 3,4). This process iterated until high levels of VE-cadherin concentration were attained and JAIL formation was blocked.

To evaluate the impact of VE-cadherin concentration on junction dynamics, VE-cadherin-EGFP was overexpressed in EC cultures (Fig. 2g) that were subjected to a scratch assay (Fig. 2h). As a result, JAIL formation, migration velocity, cell elongation and cell perimeter expansion were blocked (Fig. 2i–l; Supplementary Fig. 1a). However, the inhibitory effect was significantly less pronounced for the leader cells and the first cell rows (0–200 µm) (Fig. 2k, l) possibly because lamellipodia-mediated cell elongation at the cell junction-free side occurred in a VE-cadherin-independent manner. Since Rac activation is critical in lamellipodia formation^14^, we applied the Rac inhibitor ETH1864, which nearly totally blocked JAIL formation, cell elongation, and sheet migration (Fig. 2m–o; Supplementary Fig. 1b). Thus, we propose that the Rel-VEcad-C and Rac-dependent polarized JAIL formation, together with contractile force development^15,16^, allow directed cell migration while maintaining junction integrity.

### Polarized VE-cadherin and actin patterns in angiogenesis

**Fig. 3:**
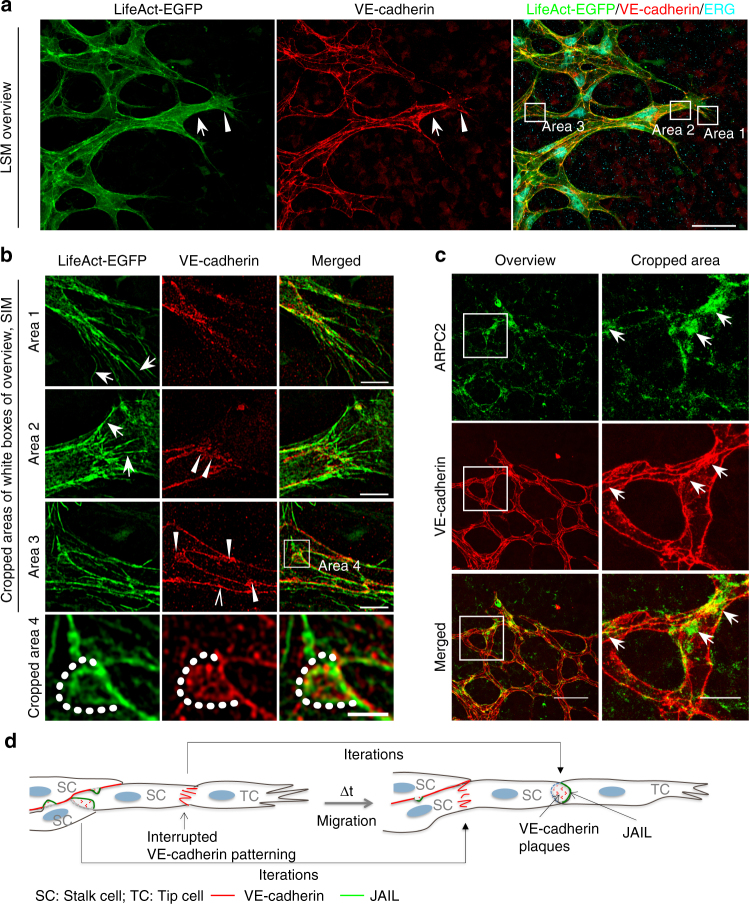
Polarized distribution of VE-cadherin and actin in sprouting ECs in developing mice retinas. **a** Laser scanning microscopy (LSM) showing an overview of the front area of a whole-mounted P6 transgenic mouse retina expressing LifeAct-EGFP additionally immune stained with anti-VE-cadherin and anti-ERG antibodies; nuclei were stained with anti-ERG. Tip cell (arrowheads) and adjacent stalk cell (arrows) are indicated. Scale bar: 40 µm. **b** High-resolution SIM of selected areas 1–3, as indicated in the right panel of **a**. Shown is one *z*-plane. (Area 1) a characteristic tip cell with large actin-based filopodia and a cytosolic spotted VE-cadherin pattern. (Area 2) A tip cell/stalk cell junction at the cell pole of elongated cells identifies terminating actin filaments (arrow) and an interrupted VE-cadherin pattern (arrowhead). (Area 3) a stalk cell/stalk cell connection. VE-cadherin plaques are indicated at the cell poles (arrowheads) and a linear VE-cadherin pattern (empty arrowhead) at lateral junctions; parallel actin filaments are also visible. Scale bar: 5 µm. (Area 4) The cropped area depicts an actin-positive JAIL (dotted line, LifeAct-EGFP) with VE-cadherin plaques (dotted line, VE-cadherin staining). Scale bar: 2 µm. **c** P7 rat retina immune labelled with ARPC2 and VE-cadherin show increased ARPC2 at the cell poles (arrows). Scale bars in the left panels and right panels represent 50 and 15 µm, respectively. **d** Scheme illustrates the iterative dynamics of VE-cadherin interruption and JAIL formation leading to VE-cadherin plaques in sprouting ECs. The VE-cadherin dynamics was particularly pronounced at the cell poles, while the lateral junctions showed moderate dynamics

Directed cell migration in angiogenesis requires cell polarisation and subcellular regulation of cell junctions. Immune staining of VE-cadherin in the developing retinas of P6 LifeAct-EGFP transgenic mice^17^ revealed VE-cadherin and actin plaque-like structures at the cell junctions (Fig. 3a). Structured illumination microscopy (SIM) disclosed a polarized distribution of both VE-cadherin and actin in tip and stalk cells (Fig. 3b), with filopodia appearing at tip cell fronts (Fig. 3b, area 1). The cell poles between tip and stalk cells or between stalk cells displayed an interrupted VE-cadherin pattern or VE-cadherin plaques, whereas the lateral junctions were characterised by a linear VE-cadherin pattern with occasional interruptions or small VE-cadherin plaques (Fig. 3b, areas 2, 3). Importantly, JAIL were visible at cell poles and at the lateral junctions (Fig. 3b). In addition, in developing yolk sac vasculature, we found a number of junction-localised VE-cadherin plaques indicative of JAIL formation, whereas in the adult vein, few VE-cadherin plaques were observed (Supplementary Fig. 2). Furthermore, in developing vessels (p7) of rat retinas, we found a prominent ARP2/3 complex signal identified by anti-ARPC2 labelling that appeared at the endothelial cell poles (Fig. 3c), suggesting an ARP2/3 complex-mediated JAIL formation at this site. The overall morphology is comparable to the morphology observed in vitro (Fig. 2a), and thus supports the concept of polarized and iterative JAIL-mediated cell junction dynamics in angiogenesis in vivo (Fig. 3d).

### VEGF induces EC elongation and migration

**Fig. 4:**
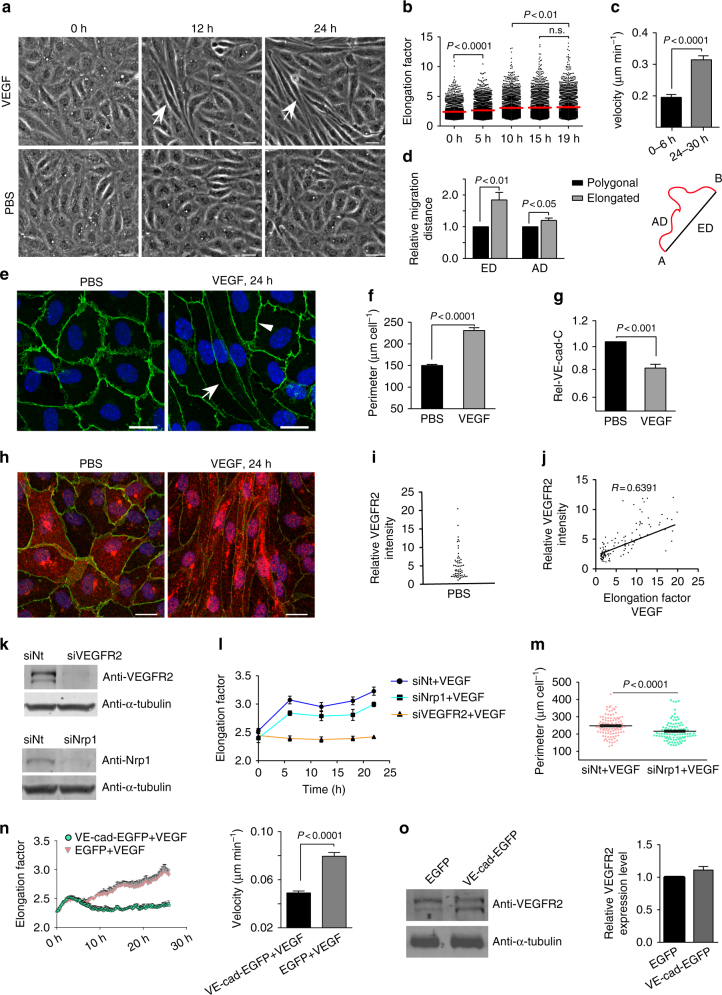
VEGFR2 and VE-cadherin expression control VEGF-induced EC elongation and migration. Confluent HUVECs were treated with 50 ng ml^−1^ VEGF or PBS. **a** Time-lapse images show cell elongation (arrows) in a subpopulation of HUVECs (Scale bar: 30 µm). **b** PhaCoB Cell Tracker analysis of cell elongation 5 h after VEGF (*n* = 3070 cells, one-way ANOVA). **c** VEGF-dependent cell velocity in the first 6 h (*n* = 71 cells) and after 24 h (*n* = 77 cells). **d** Elongated cells displayed increased 'Euclidean distance' (ED) and 'Accumulated distance' (AD) comparison to polygonal cells in the same VEGF-treated culture (*n* = 30 cells from *t* = 24–30 h, two-way ANOVA). **e** Anti-VE-cadherin-labelled HUVEC cultures. Rel-VEcad-C was decreased in a subpopulation of elongated cells (arrow) but not in polygonal cells (arrowhead) (scale bar: 20 µm). Quantification of (**f**) cell perimeter and (**g**) Rel-VEcad-C based on immunofluorescent labelling using the CBT. **h** HUVECs were stained with VE-cadherin (*green*) and VEGFR2 (*red*) (scale bar: 20 µm). **i** Quantitative distribution of the relative VEGFR2 intensity under PBS (*n* = 59 cells). **j** Correlation of relative VEGFR2 intensity with elongation factor in VEGF-treated HUVEC; *n* = 146 cells; *R* = correlation coefficient. **k** Western blot demonstrates downregulation of VEGFR2 and Nrp1 by the respective siRNAs but not non-targeting siRNA (siNt). **l** VEGFR2 downregulation totally blocked VEGF-induced cell elongation as determined by PhaCoB Cell Tracker, whereas Nrp1 downregulation had only a minor effect (*n* ≈ 3500/time point), compare Supplementary Movie 6. **m** A small but statistically significant decrease in the perimeter in Nrp1-downregulated cells quantified based on VE-cadherin-labelling using CBT (*n* = 100 cells). **n** (left) VE-cadherin-EGFP overexpression blocked the VEGF-induced elongation as analysed by PhaCoB Cell Tracker (*n* ≈ 1100 cells/time point). (right) Determination migration velocity of VE-cadherin-EGFP (*n* = 100 cells) and EGFP (*n* = 101 cells) overexpressing HUVEC in the presence of VEGF for 24 h. Compare supplementary movie 7. **o** (left) Western blot of VEGFR2 expression after VE-cadherin-EGFP overexpression. (right) Quantification of VEGFR2 expression in VE-cadherin overexpressing cells (*n* = 3). α-tubulin served as an internal loading control. Unpaired student’s *t* test was used to analyse the significance difference for experiment **c**, **f**, **g**, **m**, **n**, **o**. Error bars represent ± SEM. Representative results from three independent experiments are shown

We next investigated whether the elongation-mediated decrease in the Rel-VEcad-C increases the VE-cadherin and actin dynamics during angiogenesis. VEGF application (50 ng ml^−1^) to confluent (>10^5^ cells cm^−2^) HUVEC cultures gradually induced cell elongation in a subpopulation of the cells (Fig. 4a, b; Supplementary Movie 5). Using paired experiments, we demonstrated a relationship between cell elongation and migration activity. VEGF treatment increased cell migration after 24 to 30 h, when cells were elongated compared with the migration activity within the first 6 h (Fig. 4c). Furthermore, polarized cell migration was characterised by an increase in the Euclidean distance by 184%, and the Accumulated distance (the sum of all zig-zag movements by the cells) was increased by 119% (Fig. 4d; Supplementary Fig. 3a). By immune labelling, we also demonstrated an inverse relationship between the VEGF-induced increase in cell perimeter (153%) due to cell elongation and the decrease in the Rel-VEcad-C (down to 81%) within 24 h (Fig. 4e–g). In contrast, the total amount of VE-cadherin remained largely unchanged (Supplementary Fig. 3b, c). Consistent with the heterogeneous cell shape change after VEGF application, only a fraction of the cells displayed high VEGFR2 expression (Fig. 4h, i). In particular, the elongated cells still displayed a high level of VEGFR2 expression after VEGF stimulation, but the remaining polygonal cells did not (Fig. 4h, j). In addition, downregulation of the VEGFR2 by siRNA (Fig. 4k) totally blocked VEGF-induced cell elongation, whereas knockdown of neuropillin1 (Nrp1), a co-activator of the receptor tyrosine kinase signalling of VEGFR2^18^, had only a moderate effect (Fig. 4i, m; Supplementary Movie 6). Thus, the VEGF-induced cell elongation is regulated by VEGFR2 signalling with a minor influence of Nrp1. To evaluate whether VEGFR2-induced cell elongation and polarized migration are also dependent on a decrease in Rel-VEcad-C, VE-cadherin was overexpressed in HUVEC cultures (Supplementary Fig. 3d). Indeed, under these conditions, VEGF-induced cell elongation was largely blocked, and consequently, overall cell velocity was reduced (Fig. 4n; Supplementary Movie 7), although VEGFR2 expression remained unchanged (Fig. 4o). Thus, we conclude that VEGF-induced cell elongation requires balanced Rel-VEcad-C levels at the cell junctions to activate JAIL-mediated cell motility.

### Loss of contractility triggers VE-cadherin dynamics

**Fig. 5:**
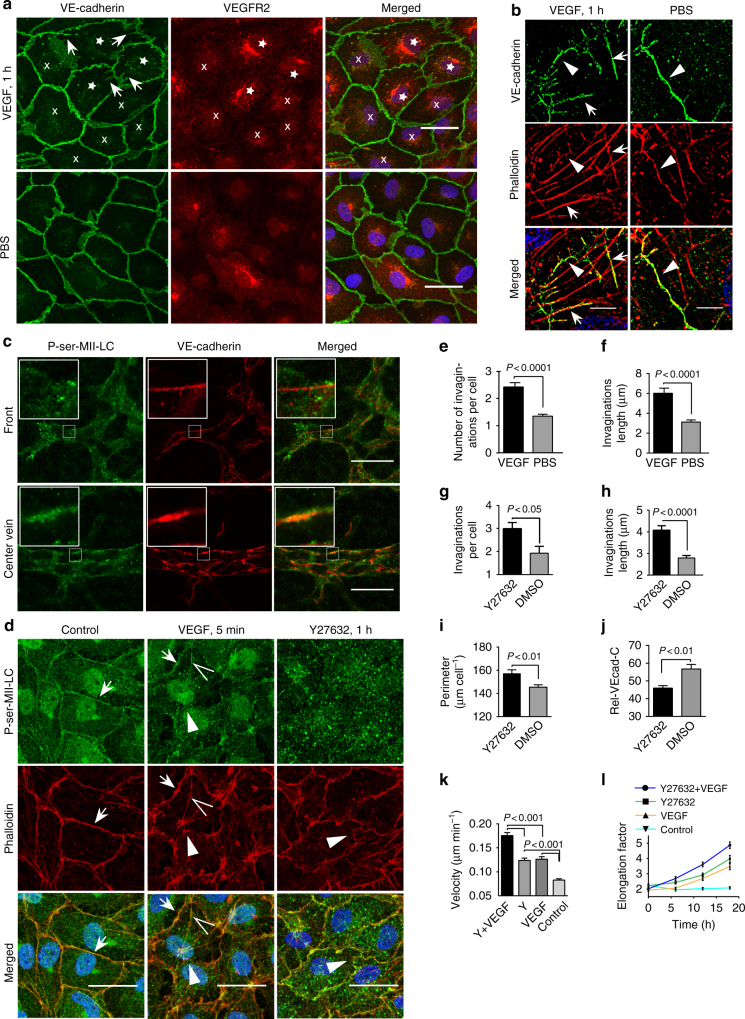
VEGF-induced shape change is accompanied by dephosphorylation of myosin II light chain at the junction. **a** LSM of VE-cadherin and VEGFR2 in HUVECs treated with 50 ng ml^−1^ VEGF or PBS. VEGF-induced invaginations (arrows) were mostly found in cells with high VEGFR2 intensity (stars) but not in less VEGFR2-expressing cells (crosses). **b** SIM of VE-cadherin- and Phalloidin-TRITC-labelled HUVECs after VEGF demonstrates that VE-cadherin invaginations largely co-localise with actin filaments (arrows). VE-cadherin and actin at cell junctions (arrowheads). **c** LSM of phospho (ser19)-myosin II light chain (P-ser-MII-LC) and VE-cadherin in P6 mice retina. P-ser-MII-LC was largely seen in the junction of centre vein but not in front ECs. **d** HUVECs treated with 50 ng ml^−1^ VEGF and 10 µM Y27632, respectively, were immune labelled with P-ser-MII-LC and Phalloidin-TRITC. VEGF induced a partial loss of P-ser-MII-LC at junctions accompanied by a partial loss of JAAF (arrowhead) and wave-like junction remodelling. Stress fibres (open arrowheads) were P-ser-MII-LC positive. Arrows indicates intact JAAF. Y27632 treatment caused a total loss of P-ser-MII-LC and also induced wave-like junction remodelling. Quantification of **e** number (*n* = 411 VE-cadherin invagination for 305 cells for PBS, 815 VE-cadherin invaginations for 338 cells for VEGF) and **f** length of VE-cadherin invaginations in VEGF- (*n* = 98 VE-cadherin invaginations) and PBS-treated (*n* = 92 VE-cadherin invagination) cells. Quantification of (**g**) VE-cadherin-positive invaginations (*n* = 422 and 280 VE-cadherin invaginations for 143 Y27632 and DMSO treated cells respectively), (**h**) invagination length (*n* = 227 and 232 VE-cadherin invaginations for Y27632 and DMSO separately), (**i**) cell perimeter, and **j** Rel-VEcad-C in Y27632-treated cells for 1 h or DMSO based on VE-cadherin immune labelling (*n* = 143 cells); (compare Supplementary Fig. 5b). Unpaired student’s *t* test was used for (**e**–**j**). **k**, **l** HUVECs pre-treated with 10 µM Y27632 for 1 h were applied with VEGF for another 18 h. Quantification of (**k**) migration velocity (*n* = 100 cells) and (**i**) elongation factor (87, 97, 87 and 87 cells were used for Y27632+VEGF, Y27632, VEGF, and control, respectively) using Fiji (compare Supplementary Fig. 5c); one-way ANOVA. Y = Y27632. Error bars indicate ± SEM. Scale bars represent 30 µm in **a**, **c**, **d** and 5 µm in **b**

VEGF-activated cell junction dynamics was evaluated in VE-cadherin-EGFP-expressing confluent HUVEC cultures by spinning disc microscopy (SDM). Time-lapse recordings revealed increased VE-cadherin dynamics within 10 to 20 min after VEGF application in a subset of cells (Supplementary Movie 8). In particular, VE-cadherin remodelling accompanied the appearance of spatiotemporally restricted invaginations that increased in number and length within 1 h and preferentially appeared in cells with high VEGFR2 expression (Fig. 5a, b, e, f). SIM of VEGF-treated HUVECs revealed a reduced co-distribution of VE-cadherin and actin at cell junctions and invaginations as well as a moderate amount of stress fibre formation (Fig. 5b). Actin-driven junction dynamics were upregulated shortly after VEGF application. Furthermore, a few vesicle-like structures were released from the junctions, resembling endocytic processes (Supplementary Movie 8). However, the VE-cadherin-positive vesicle-like structures that were taken up were largely negative for the endosome antigen 1 (EEA1) and Rab5 (Supplementary Fig. 4). For this reason, no further investigations were carried out, since we expected this small number of endocytic vesicles to play only a minor role in the phenomena observed here.

We next investigated whether myosin light chain (MLC) phosphorylation, which controls actin/myosin II-mediated tension development in endothelium, might play a role in actin and VE-cadherin dynamics^19,20^. Immune labelling of confluent cell cultures and developing retinas in vivo with a specific phospho-(ser19)-myosin II light chain antibody (P-ser-MII-LC) produced a clear signal at cell junctions of centre vein ECs in vivo (Fig. 5c) and in confluent HUVEC cultures (Fig. 5d). The signal was largely negative at front EC junctions in the retina (Fig. 5c) and at the junctions of HUVECs within 5 min after VEGF application (Fig. 5d). This phenomenon was accompanied by a partially (local) dissociated JAAF and stress fibre formation that were positive for P-ser-MII-LC in HUVEC cultures, which is in agreement with the appearance of wave-like junction remodelling (Fig. 5d). In addition, a complete loss of P-ser-MII-LC was evident 1 h after application of the ROCK inhibitor Y27632, which blocks actin/myosin II-mediated contraction^21,22^ (Fig. 5d). VE-cadherin immune labelling as well as live-cell imaging revealed a dramatic increase in the size and number of invaginations as well as the appearance of curved VE-cadherin junctions after Y27632 application (Fig. 5g, h; Supplementary Movie 9; Supplementary Fig. 5a, b), Furthermore, an increase in junction length and a decrease in Rel-VEcad-C (Fig. 5i, j; Supplementary Movie 9) accompanied cell migration and cell elongation within 18 h (Fig. 5k, l; Supplementary Fig. 5c). A synergistic effect of Y27632 and VEGF was evident after combined application (Fig. 5k, l). Thus, we propose a relaxation-dependent subcellular activation of actin dynamics that initiates cell elongation and polarisation and increases migration activity.

### Microtubule dynamics is key in polarized cell elongation

**Fig. 6:**
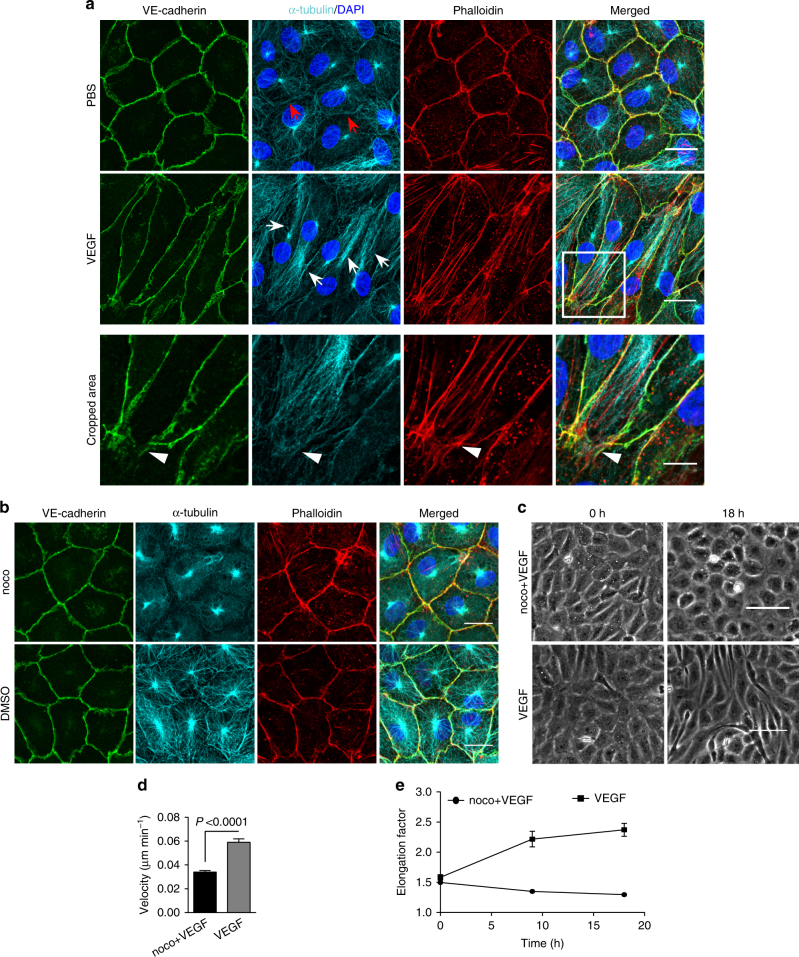
Microtubules (MT) are indispensable for VEGF-induced cell elongation. **a** Confluent HUVECs immune labelled for VE-cadherin, α-tubulin and Phalloidin-TRITC after VEGF treatment for 24 h or PBS for control, as indicated. Nuclei are stained blue with DAPI. LSM demonstrates MT in control cells evenly distributed throughout the cells, while a few MT are aligned in parallel with JAAF (red arrows). VEGF-induced elongated cells display MT running parallel to the longitudinal cell axis together with stress fibres, and MT are enriched at the leading edge (white arrows; for dynamics compare supplementary Movie 10) (Scale bar: 20 µm). The cropped area displays an interrupted VE-cadherin pattern, MT enrichment, and stress fibres at the cell poles (arrowheads; for dynamics compare Supplementary Movies 10 and 11) (scale bar: 10 µm). **b** Confluent HUVEC cultures treated with 50 ng ml^−1^ nocodazole for 4 h and subsequently labelled with VE-cadherin antibody and with Phallodin-TRITC for actin filaments. MT depolymerisation had less effect on the JAAF and VE-cadherin distribution (Scale bar: 20 µm). **c**–**e** Confluent HUVECs pre-treated with 50 ng ml^−1^ nocodazole for 30 min and then treated with VEGF for another 18 h. **c** Phase-contrast microscopy revealed that nocodazole inhibited VEGF-induced cell elongation (scale bar: 80 µm). Quantification of (**d**) cell velocity and (**e**) cell elongation using Fiji software (100 cells were analysed at *t* = 0, 100 cells at *t* = 9 h, 81 cells at *t* = 18 h for nocodazole+VEGF treatment; and 100 cells were analysed at each time point for VEGF treatment, unpaired student’s *t* test). noco: nocodazole. Representative results from three independent experiments are shown. Error bars indicate ± SEM

Cell polarisation has been shown to involve microtubules (MT) activity^23,24^. Labelling of MT in HUVECs documented a single MT organization centre (MTOC) from which MT distributed throughout the cells; a few MT were enriched at cell junctions, where they paralleled the JAAF filaments and VE-cadherin (Fig. 6a). Twenty-four hours after, VEGF application, the MT were distributed along polarized stress fibres, and this was accompanied by an interrupted VE-cadherin pattern at the cell pole (Fig. 6a). Analysis of MT dynamics was performed in β5-tubulin-EGFP-expressing HUVEC cultures by SDM. The addition of VEGF resulted in increased MT dynamics, especially in the cell’s half containing the MTOC. A progressive increase in polarized MT dynamics ultimately led to cell elongation and increased migration (Fig. 6a, Supplementary Movie 10). MT depolymerisation by 50 ng ml^−1^ nocodazole caused a loss of cell polarity, elongation, and migration and led to rounding of the cells within 2 h (Supplementary Movie 10), while VE-cadherin was still localised at the junction after 4 h (Fig. 6b). Furthermore, rounded cells still displayed protrusions that appeared in a randomised manner (Supplementary Movie 10). Similar results were obtained when HUVEC cultures were treated with nocodazole prior to VEGF application (Fig. 6c–e). The results are consistent with the nocodazole-mediated inhibition of shear stress-induced cell elongation^25^. These data demonstrate that MT activity is indispensable for initiation and maintenance of VEGF-induced endothelial shape change and for polarized cell dynamics but not for maintenance of VE-cadherin-mediated cell adhesion and dynamics.

### Cell junction dynamics in VEGF-induced elongated cells

**Fig. 7:**
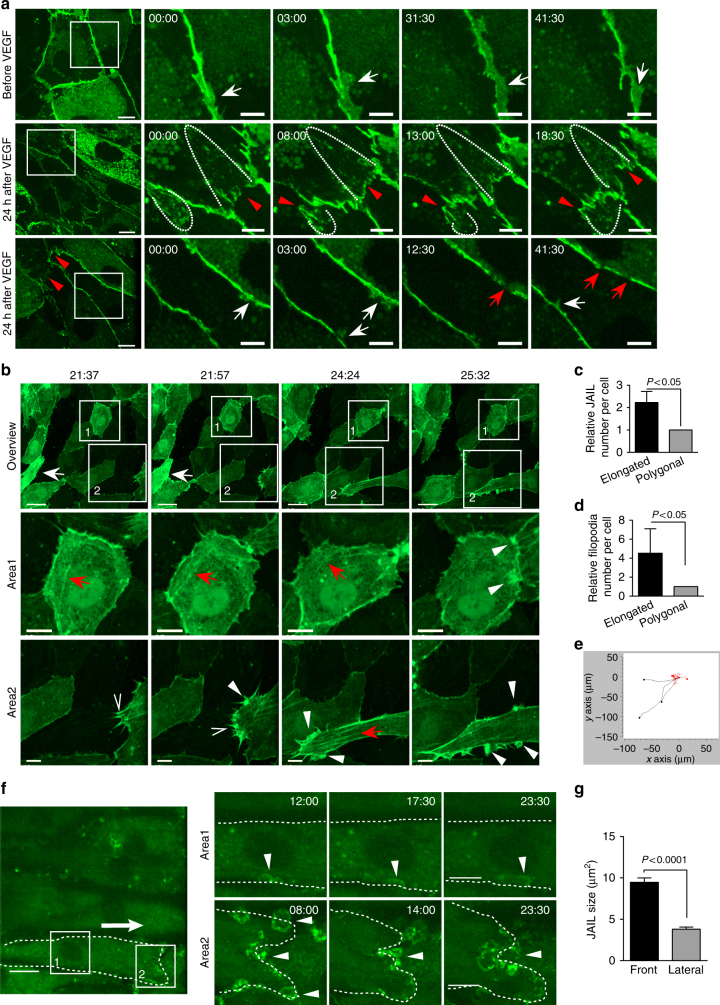
Junction dynamics upon VEGF treatment in confluent HUVECs. HUVECs expressing (**a**) VE-cadherin-EGFP (**b**–**e**) LifeAct-EGFP or (**f**–**g**) EGFP-p20, respectively, were treated with 50 ng ml^−1^ VEGF. **a** (upper) Controls display regular VE-cadherin-EGFP dynamics with VE-cadherin plaques (white arrows) due to JAIL formation. (middle) After VEGF treatment, elongated cells exhibit a polar distribution with an interrupted VE-cadherin pattern at cell poles (red arrowheads) followed by transient formation of new large VE-cadherin plaques (dotted lines). (lower) Lateral junctions display linear VE-cadherin pattern with appearance of small VE-cadherin plaques (white arrows). Small VE-cadherin interruptions (red arrows) appear transiently. Time scale: mm:ss; scale bars indicate 10 and 5 µm in the overview and cropped images. **b** LifeAct-EGFP dynamics after VEGF. (upper) The overview images demonstrate both polygonal cells and elongated cells (white arrows) (scale bar: 30 µm). (Area 1) Polygonal cells display constitutive junction remodelling that includes the transient appearance of small stress fibres (red arrows) and small JAIL (white arrowheads) without significant cell elongation. (Area 2) A progressively moving elongated cell exhibits many filopodia (empty arrowheads) that appear on the sides of large JAIL (white arrowheads). Note: stress fibres (red arrows) preferentially emerge at the leading edge of the cells. Time scale: hh:mm; scale bar: 10 µm. **c**, **d** JAIL and filopodia number in polygonal and elongated cells in VEGF-treated culture. **e** Track plots illustrate migration path of elongated cells (black line) and polygonal cells (red line). **f** Small JAIL formation at the lateral junctions (cropped area 1, arrowheads) and large JAIL development at the cell poles (cropped area 2, arrowheads) in VEGF-induced elongated ECs expressing EGFP-p20. The dotted line indicates the cell border; arrow indicates the direction of cell movement. Time scale, mm:ss; scale bar represent 20 and 10 µm in overview and cropped images. **g** JAIL size at the cell poles and the lateral sides of elongated cells induced by VEGF. 157 JAIL at the cell pole and 152 JAIL at the lateral junctions from three independent experiments were analysed over a period of time (30 min). Unpaired student’s *t* test was used for statistics analysis and error bars indicate ± SEM

SDM of HUVECs that moderately express VE-cadherin-EGFP, LifeAct-EGFP or EGFP-p20 was performed to investigate VEGF-induced junction remodelling. Under control conditions, VE-cadherin-EGFP displayed a moderate but constitutive formation of plaque-like structures that correspond to JAIL (Fig. 7a). VEGF-induced cell elongation established a planar polarity with two short cell poles, one of which represents the leading and the other the trailing end; both displayed an interrupted VE-cadherin–EGFP pattern that alternates with VE-cadherin plaque formation induced by large JAIL. A faint, linear VE-cadherin-EGFP pattern developed at lateral junctions with the appearance of much smaller VE-cadherin plaques (Fig. 7a; Supplementary Fig. 6a). Immune labelling revealed that only 43% of the elongated cells displayed an interrupted VE-cadherin pattern at the cell poles at a given time point (Supplementary Fig. 6b), which is consistent with rapid constitutive VE-cadherin dynamics and disintegration of the cell junctions during cell migration.

Actin dynamics was studied in LifeAct-EGFP-expressing confluent HUVEC cultures. Application of VEGF induced a polarized, progressive cell elongation in some cells that constantly migrated in one direction and displayed large JAIL and filopodia formation, whereas polygonal cells displayed slow and randomised movement (Fig. 7b–e; Supplementary Movie 11). Formation and retraction of filopodia seemed to occur spontaneously at cell poles (Supplementary Movie 11), whereas JAIL was followed by formation of new VE-cadherin adhesion sites (Supplementary Movie 3). Filopodia rarely developed at the lateral junctions of elongated cells, whereas small JAIL appeared regularly, probably contributing to the relative cell migration. Furthermore, the local loss of JAAF accompanied polarized migration activity at the side of the cells where the stress fibres appeared, a result consistent with MT distribution and dynamics.

Since stress fibres are contractile structures in endothelium^20,26^, we proposed that newly formed VE-cadherin adhesion sites at the leading end might function as anchors for migrating cells to be pulled by stress fibre contraction. This process was indeed visible during formation of large VE-cadherin adhesion plaques at the cell poles during polarized cell migration (Fig. 7a; Supplementary Fig. 6a), the data that are consistent with the recently reported development of contractile forces at endothelial junctions during sheet migration^15^. In contrast, polygonal cells still retained the JAAF band and did not develop stress fibres or show increased actin dynamics, irrespective of the presence of VEGF (Fig. 7b; Supplementary Movie 11). Polarized JAIL dynamics were further studied by live-cell imaging using EGFP-p20-labelled HUVEC cultures, which confirmed the high activity and formation of large JAIL at the leading end (Fig. 7f; Supplementary Movie 12). These JAIL were about 2.5 times larger than those at the lateral junctions (Fig. 7g). The data document that directed migrating elongated cells, but not randomly migrating polygonal cells, display a high degree of polarized junction dynamics and the establishment of a polarized VE-cadherin, MT, and actin patterning suggests that cell elongation is a key parameter in cell motility.

### Rac1 activation triggers cell elongation and JAIL formation

**Fig. 8:**
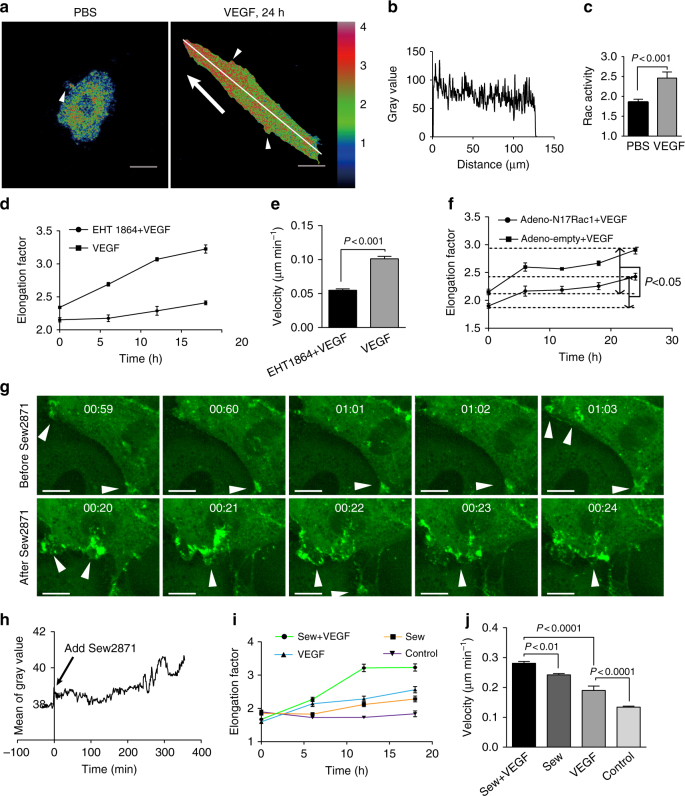
Polarized Rac activity is required for VEGF-induced cell elongation and migration. **a–c** Confluent HUVECs expressing FRET sensor Raichu-Rac1 were treated with VEGF for 24 h. **a** Representative images showing different levels of Rac1 activity within control cells and VEGF-induced elongated cells. Control cells displayed less and randomised Rac1 activity (arrowhead; compare Supplementary Movie 13). Polarized, higher Rac1 activity is observed at the leading front (arrow indicates migration direction; compare Supplementary Movie 13). Small foci of elevated Rac activities are observed at the lateral side (arrowheads); scale bars: 20 µm. **b** Plot of fluorescence intensity along the line is indicated. **c** Quantification of overall Rac1 activity as defined by the ratio of YFP/CFP in PBS- or VEGF-treated cells (*n* = 74 and 87 cells were analysed from 4 independent experiments for PBS and VEGF, respectively (unpaired student’s *t* test)). **d**, **e** Confluent HUVECs pre-treated with EHT1864 for 30 min were treated with VEGF for additional 19 h. Quantification of (**d**) elongation factor and (**e**) migration velocity after EHT1864 + VEGF (*n* = 100 cells) and VEGF (*n* = 101 cells) treatment for the time period of 19 h (unpaired student’s *t* test). **f** Confluent HUVEC cultures expressing adeno-N17Rac1 display decreased VEGF-induced cell elongation as determined by PhaCoB Cell Tracker (*n* ≈ 1000cells/time point). **g**–**h** Sew2871 application upregulated VEGF-induced cell elongation and migration ability. **g** Time-lapse recordings of confluent HUVECs expressing EGFP-p20 treated with 10 µM Sew2871. Sew2871 largely increased JAIL formation (arrowheads). Time scale: hh:mm; scale bars: 10 µm. **h** Quantification of overall EGFP-p20 intensity upon Sew2871 application. **i**,** j** Confluent HUVEC cultures were pre-treated with 10 µM Sew2871 for 1 h before 50 ng ml^−1^ VEGF was applied. **i** Quantification of elongation factor over time, as indicated (data are based on the analyses of 173, 162, 164, 173 cells for *t* = 0, 6, 12 and 18 h, respectively). **j** Comparison of migration velocity after Sew2871+VEGF, Sew2871, VEGF and control treatments, respectively, for 16 h (data are based on analyses of 103, 102, 99 and 100 cells for each group). One-way ANOVA was used to analyse significance difference for **i**, **j**. Representative results from three independent experiments are shown. Error bars represent ± SEM

RhoGTPases Rac are critical in directed cell migration^14^, and VEGFR2 activation targets the small GTPase Rac1^27,28^. Our observation of VEGF-induced polarized actin dynamics at EC junctions led us to suggest a role for Rac in this process. Expression of a FRET-based Rac sensor^29,30^ in confluent HUVECs revealed randomised Rac activity at cell junctions in control cells, at sites of small actin-driven JAIL.  In contrast, VEGF-treated elongated cells, embedded in a confluent cell layer, exhibited elevated levels of Rac1 activity at the leading end where large JAIL appeared (Fig. 8a–c; Supplementary Movie 13). A small amount of spatiotemporally restricted elevation of Rac appeared at lateral junctions, consistent with the presence of small JAIL at this site (Fig. 8a–c; Supplementary Movie 13).

Application of the Rac1 inhibitor EHT1864 or expression of the dominant negative N17Rac1 blocked VEGF-induced cell elongation and migration (Fig. 8d–f; Supplementary Fig. 7a, b; Supplementary Movie 14). Interestingly, application of EHT1864 to LifeAct-EGFP-expressing HUVEC cultures inhibited JAIL but not filopodia formation (Supplementary Fig. 7c; Supplementary Movie 14), suggesting that filopodia were not required to drive cell elongation. In line with this, stimulation of Rac activity via application of 10 µM Sew2871, a spingosine-1-phosphate receptor (S1P-R) activator, significantly increased JAIL formation, and a synergistic effect of Sew2871 and VEGF on cell elongation and migration velocity was observed (Fig. 8g–j; Supplementary Fig. 7d; Supplementary Movie 15).

### ARP2/3 complex controls JAIL formation in angiogenesis

**Fig. 9:**
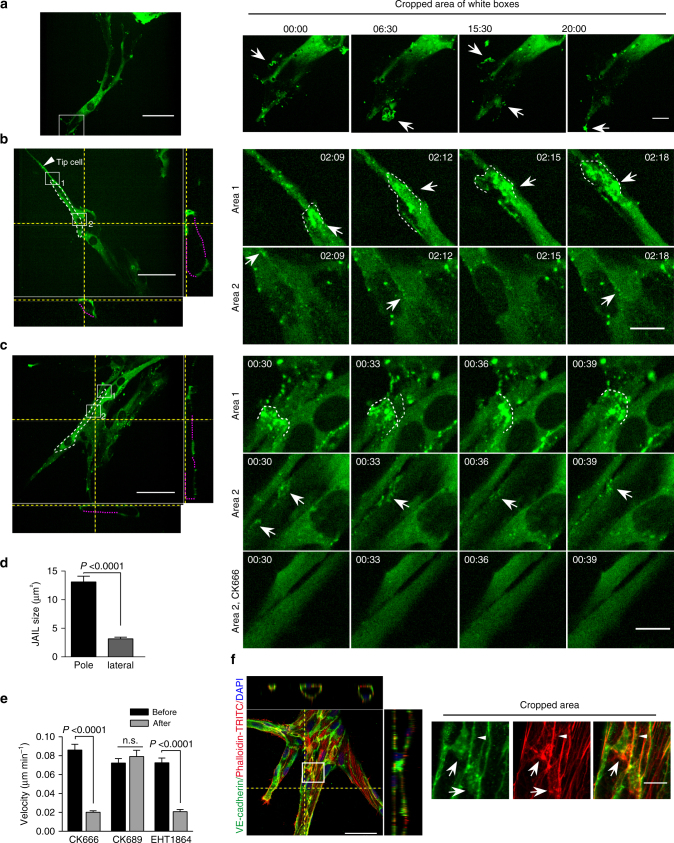
Polarized JAIL dynamics during sprouting angiogenesis in fibrin angiogenesis assay using EGFP-p20 expressing HUVECs. **a**–**c** SDM based time-lapse recordings (TLR) of different cell junctions during sprouting angiogenesis. In the overview of **b**, **c**, **f**, Yellow dotted lines indicate the *z*-projection level. Purple dotted line completes the lumen-forming sprouts since not all cell expressed the fluorescence-tagged protein and white dotted line outlines the cell junctions. **a** Overview of tip cell sprouts. (cropped area) EGFP-p20-positive plaques identified transient lamellipodia and filopodia at the tip cell front (arrows). **b** Overview and Z-projection of tip cell/stalk cell junctions. (cropped area) TLR of large JAIL that development at the cell poles (white dotted lines, area 1) and faint and small JAIL appearing at the lateral junction (arrows, area 2). **c** JAIL formation between stalk cell/stalk cell junctions. (cropped area 1) TLR demonstrate large JAIL formation at the cell pole (dotted line). (cropped area 2, middle panel) Small JAIL developed at lateral junction (arrows). (cropped area 2, lower panel) ARP2/3 complex inhibitor CK666 applied to the same cells blocked JAIL formation. **d** Quantification of JAIL size in the migrating pole and lateral side of the sprouting ECs. Sixty-four JAIL at the leading cell pole and 45 JAIL at the lateral junctions from 3 movies over a period of 30 min were analysed, unpaired student’s *t* test. **e** Inhibition of cell migration ability after ARP2/3 complex inhibitor (CK666) and inactive control inhibitor (CK689) and Rac inhibitor EHT1864. Quantification is based on phase-contrast time-lapse recordings for the time period up to 6 h; *n* = 67, 70, 82 cells before and 69, 70, 85 cells after CK666, CK689, or EHT1864 treatment, respectively. Cells were pooled from two independent experiments; two-way ANOVA. **f** Overview and Z-projections of vessel sprouts in fibrin angiogenesis assays 5 days after seeding; cells were fixed and labelled with Phalloidin-TRITC and VE-cadherin. (cropped areas) JAIL are indicated at cell poles by the appearance of large VE-cadherin plaques (arrows) that co-localise with the actin network (arrows), whereas small VE-cadherin plaques appear at lateral junctions (arrowhead). Error bars represent ± SEM; scale bars indicate 50 and 10 µm in the overview and cropped areas, respectively

The data presented thus far strongly suggest that polarized cell contact dynamics involving loss of contraction, MTs, ARP2/3 complex, and actin and VE-cadherin dynamics provide the mechanistic preconditions for cell elongation and directional cell migration in angiogenesis. For further evaluation, we used an in vitro angiogenesis model utilising HUVEC-covered micro-carrier beads embedded in fibrin gel^31,32^. Phase-contrast time-lapse recordings revealed endothelial outgrowth from the bead surface, with a tip cell-like morphology and long filopodia and lumen formation (Supplementary Movie 16, left panel). Leader cells and followers interconverted at the leading position (Supplementary Movie 16, left panel), as described previously^2^. Furthermore, 4D time-lapse recordings of EGFP-p20-expressing HUVECs demonstrated EGFP-p20-positive filopodia-like structures and lamellipodia at the front end of migrating tip cells (Fig. 9a), which is consistent with the demonstration that lamellipodia as well as filopodia can guide tip cells^33^. To better visualise the junction activity in tube-forming sprouts (Supplementary Movie 16, right panel), we examined a *z*-plane that was close to the tube wall (Supplementary Movie 17, 18) that allowed us to follow the junction dynamics at cell poles and lateral junctions. Both tip cell/stalk cell junctions and stalk cell/stalk cell junctions displayed high ARP2/3 activity at the cell poles, forming large JAIL as verified by the curved, overlapping EGFP-p20-positive structures, whereas the lateral junctions displayed less EGFP-p20 activity, indicative of small JAIL (Fig. 9b, c; Supplementary Movie 17, 18). JAIL were about 3.5 times larger at cell poles than at lateral junctions (Fig. 9d). The application of the ARP2/3 complex inhibitor CK666 or the Rac inhibitor EHT 1864 blocked JAIL formation (Fig. 9c) and reduced the migration velocity (measured by nuclear movement) in the 4D angiogenesis assay to about 25% of control values (Fig. 9e). We also observed stalk cell mitosis, which took place in a lumen-forming vessel. After nuclear division, cytokinesis displayed EGFP-p20 at the contractile ring, which is in accordance with the appearance of ARP2/3 complex in dividing yeast^34^. After cytokinesis, elongated daughter cells showed EGFP-p20 accumulation at their poles opposite to the plane of division, suggesting formation of large JAIL at these sites (Supplementary Movie 18). Consistent with this, immune labelling confirmed large VE-cadherin plaques and actin networks at the cell poles, whereas small VE-cadherin plaques and actin networks were displayed at the lateral junctions (Fig. 9f). The impact of the ARP2/3 complex was also investigated in a Matrigel angiogenesis assay using the ARP2/3 complex inhibitors CK548 and CK666; these inhibitors completely blocked the formation of tube-like structures, whereas the inactive analogues CK312 or CK689 had no effect (Supplementary Fig. 8). Furthermore, removing the active inhibitor CK548 re-established the formation of tubule structures (Supplementary Fig. 8). The data demonstrate that the ARP2/3 complex is essential for tubule formation.

### Inhibition of Notch signalling-induced cell elongation

VEGFR2 signalling and the Notch pathway control each other during angiogenesis^35,36^. Pharmacological inhibition of Notch by administration of the γ–secretase inhibitor DAPT led to increased sprouting angiogenesis in wild-type mice (Supplementary Fig. 9a), as previously described^37^, with increased perimeter of ECs in the sprouts (Supplementary Fig. 9b). These data were confirmed in endothelial-specific *DLL4* knockout mice that also displayed upregulated angiogenesis (Supplementary Fig. 9c); however, the total amount of VE-cadherin remained constant upon inhibition of Notch signalling, as verified by RT-PCR (Supplementary Fig. 9d). The results are in accordance with the observation that a deletion of Rbpj, coding for a principal transcriptional effector of Notch signalling, increases EC size in mouse retinas^38^. Application of DAPT to HUVEC cultures caused cell elongation (Supplementary Fig. 10a, b), increased the cell perimeter, and decreased the Rel-VEcad-C (Supplementary Fig. 10c, d), which was accompanied by a decrease in barrier function due to cell elongation (Supplementary Fig. 10e). DAPT application did not change the expression of cell adhesion molecules (Supplementary Fig. 10f, g) but downregulated Hes-1 and DLL4, which confirms the DAPT inhibitory activity (Supplementary Fig. 10h). Furthermore, cell migration increased in a scratch assay of HUVEC cultures (Supplementary Fig. 10i, j). Taken together, these data provide additional evidence that cell elongation is a critical parameter for determining Rel-VEcad-C and migration activity.

## Discussion

Our data document cell elongation is required for initiation and maintenance of polarized cell migration during angiogenesis. We showed that the elongation of polygonal cells in confluent cultures leads to an increase in the cell contact length and to a reduced Rel-VEcad-C. In contrast the total VE-cadherin concentration remains unchanged as verified in ECs of vessel sprouts in wt and in DAPT-treated mice in vivo. These data are in agreement with VE-cadherin-dependent stalk cell elongation in zebrafish^39^, and with an unchanged VE-cadherin expression in growing and confluent HUVEC cultures^8,40^, an unchanged VE-cadherin mRNA expression, and an increase in cell size or cell elongation in angiogenesis, as shown in vivo and in vitro^38,39,41,42^.

The here presented data could explain why a moderate VE-cadherin depletion increases sprouting in vitro^6^ and in the murine retina in vivo^3^. In contrast to VEGF, which does not change VE-cadherin expression, elongated arterial ECs and shear stress-exposed EC have an upregulated VE-cadherin expression that compensates for the increase in cell junction length^43^. In addition, ablation of the *Cdh5* gene in mouse embryos alters vessel growth and lumen formation^7,44^. Taken together, the data indicate a balanced VE-cadherin concentration at the cell contacts is decisive for induction and progression of angiogenesis. A decrease in Rel-VEcad-C is a stimulus for actin-mediated JAIL formation under the guidance of the WAVE/WASP/ARP2/3 complex, since a carboxyl-terminal VE-cadherin deletion mutant results in increased JAIL formation^8^, whereas VE-cadherin overexpression blocks JAIL formation, VEGF-induced cell elongation, and cell migration. We propose that the different VE-cadherin patterns, particularly noticeable in the VEGF-induced elongated cells, are a critical subcellular determinant of polar cell migration. We suggest that the Rac-dependent formation of large JAIL, appearing at the cell poles with an interrupted VE-cadherin pattern, drives the forward-directed migration, whereas small JAIL at the lateral cell junctions that form a linear VE-cadherin pattern allow the relative movement of adjacent cells. This is different from highly dynamic subconfluent cultures, which also have large cell perimeters but show a heterogeneous VE-cadherin pattern and a randomised JAIL formation and cell migration, indicating the importance of the elongated cell shape for polarized cell migration. In fish embryos, a transient shape change has been attributed to arteriogenesis^45^ and seems to be related to Notch controlled CXCR4 expression^46^ that in turn was shown to control VE-cadherin expression^47^. Importantly, blood flow induces inverse blebs at the apical EC membrane that drives lumen formation^48^ and might involve JAIL dynamics, a basal-to-apical VE-cadherin flow^49^, an actomyosin flow^50^, and force development of protrusions^51^ may also play a role.

The VEGF-induced cell elongation is dependent on a high VEGFR2 expression, which occurs only in a subpopulation in HUVEC cultures and thus could explain the heterogeneous cell elongation after VEGF stimulation. These data are in line with results of recent studies that suggest a salt-and-pepper pattern of VEGFR2 expression in sprouting ECs^52,53^. VEGF-mediated signalling appears to induce a polarized modulation of cell junctions to induce polarized JAIL formation and thus cell elongation. In particular, we showed that MLC dephosphorylation triggers the dissociation of JAAFs, and that VEGF treatment or ROCK inhibition results in the formation of VE-Cadherin-positive invaginations. These invaginations might dilute the Rel-VEcad-C locally and thus allow JAIL formation, leading to cell elongation and directed cell migration. Recent work performed in fish embryos indicated that actin polymerisation rather than contraction mediates stalk cell elongation, while application of contraction inhibitors caused defects in VE-cadherin and disorganised actin^39^. These junctional defects are comparable to both the VEGF-induced VE-cadherin remodelling and after inhibition of ROCK, which we here identified as a stimulus for cell elongation due to JAIL formation. This mechanism might also account for shape change during arteriogenesis^46^. In some cases, we observed that vesicle-like structures were pinched off from invaginations but were not further processed in a classical degradation process (Rab5 and EEA1 negative). VEGF was proposed to cause rapid VEGFR2 turnover and induce signalling for tip cell formation as well as VE-cadherin endocytosis^53,54^, but this process does not appear to account for a significant decrease in Rel-VEcad-C. However, previous work^55^ and this study demonstrated VEGF-induced VE-cadherin invaginations that might behave differently in biochemical and endocytic pathways.

Both cell polarisation and migration require polarized and constitutive MT dynamics, as application of nocodazol not only blocks VEGF-induced cell elongation and directed migration but also converts elongated cells into polygonal ones. VEGF-induced cell elongation and increased MT activity occur preferentially at the MTOC-localised cell half, indicating a critical role of MT and MTOC in initiation and maintenance of cell polarisation in the endothelium; these results are in agreement with previous reports (for review see^24,56^). In addition, Rac activity is critical for the induction and maintenance of VEGF-induced cell elongation and migration, during which a MT-dependent delivery of Rac to the site of action might take place^23,27,28,57^. In addition, depletion of α-parvin, an actin-binding protein, inhibits Rac activity^58^, which is in turn a potent activator of JAIL and angiogenesis^59^.

**Fig. 10:**
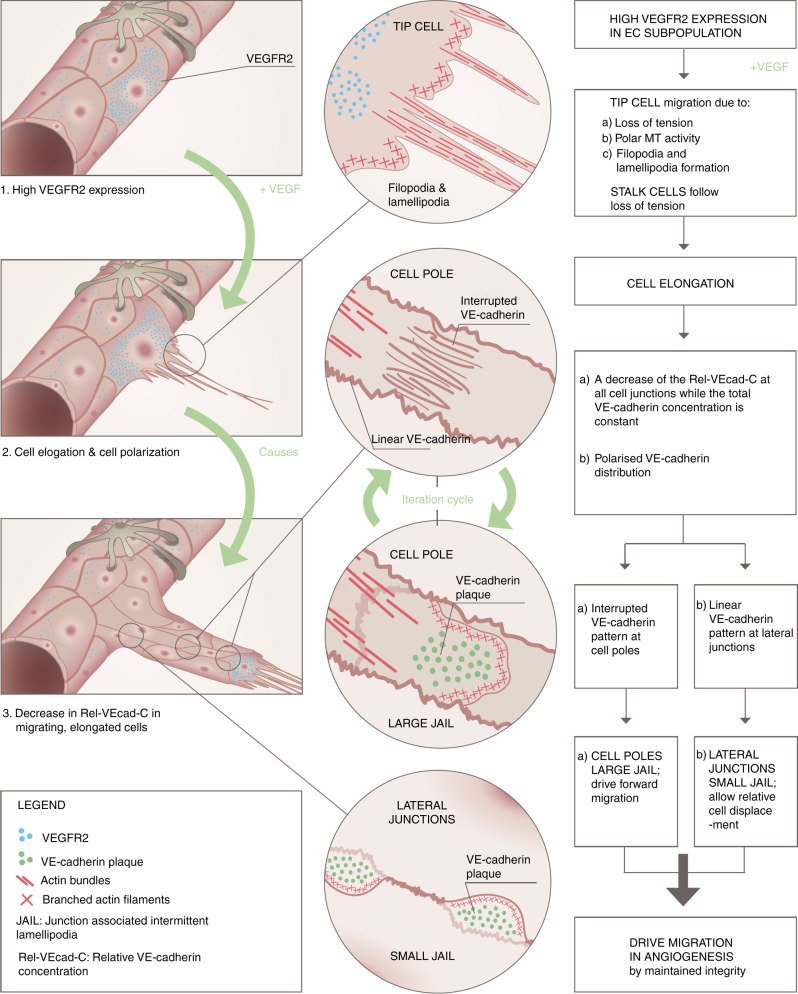
The scheme illustrates the proposed model of the molecular mechanisms driving cell migration in angiogenesis

Directed cell migration occurs at the leading cell pole, which mostly has an interrupted VE-cadherin pattern, and induces the formation of large JAIL. As a result of JAIL formation, extensive VE-cadherin plaques are formed, which can act as adhesion anchors and allow the forward migration of the cell. The stress fibres at the leading edge support the directed migration by contraction, a process recently described in detail^15^. Although filopodia were frequently seen in angiogenesis, as described^33^, they could not be blocked by ARP2/3 complex inhibition and might, therefore, have more of a sensory function^1^. Furthermore, filopodia were recently suggested to be an intermediate phase of VE-cadherin-mediated cell contact formation^60^, which is in agreement with the JAIL-mediated VE-cadherin dynamics. The junctional regulation by JAIL and the MTOC-dependent cell polarisation described here may also explain the backward migration of cells, as observed in cell culture models and in zebrafish in vivo^2^. Furthermore, JAIL formation is an iterative process as long as the local VE-cadherin concentrations are below a threshold level, and JAIL formation declines if the VE-cadherin concentration increases. Thus, JAIL formation and shape change might also significantly contribute to vessel extension, lumen formation, and termination of angiogenesis. We assume that the relaxation mediated cell elongation is a relatively simple and for the cell energy-saving possibility to increase the junction dynamics and thus allows migration activity. The concept of cell contact regulation in angiogenesis presented here describes a mechanism to control the polarized cell contact dynamics and cell migration. (Fig. 10).

## Methods

### Reagents

Recombinant VEGF165 (VEGF) was obtained from R&D Systems (#293-VE-010 Wiesbaden-Nordenstadt, Germany) and diluted in phosphate-buffered saline (PBS) containing 0.1% bovine serum albumin (BSA). The Rac inhibitor (EHT1864) and microtubule polymerisation inhibitor (nocodazole) were obtained from Santa Cruz Biotechnology (#sc-361175, Heidelberg, Germany) and Sigma (#M1404, Munich, Germany), respectively. Sphingosine-1-phosphate 1 receptor (S1PR1) activator (Sew2871) was provided by Abcam (#ab120983, Cambridge, UK). The γ–secretase inhibitor (GSI-IX, LY-374973, N-[N-(3,5-difluorophenacetyl)-L-alanyl]-S-phenylglycine t-butyl ester, DAPT, #565770), and the ARP2/3 complex inactive inhibitors CK312 (#182518) and CK689 (#182517) and active inhibitor CK666 (#182515) were purchased from Calbiochem (Bad Soden, Germany), and the active inhibitor CK548 was purchased from ChemDiv (#K205-1650, San Diego, USA). Regents were used at concentrations as indicated in the text.

### Cell culture

Human umbilical vein ECs (HUVECs) were isolated from umbilical cord veins of different donors, a procedure that was approved by the ethics committee of the WWU- Muenster at the request of the Institute of Anatomy and Vascular Biology (2009-537-f-S). Umbilical cords veins were perfused with PBS (#21600069, Thermo Fisher Scientific, Waltham, MA, USA) followed by digestion with 1 mg ml^−1^ Collagenase (#C2-22, Biochrom, Berlin, Germany) diluted in PBS for 10 min at 37 °C in a water bath. The detached cells were collected and centrifuged at 200 × g. Cell were cultured in Endothelial growth medium (PromoCell, Heidelberg, Germany) supplemented with Endothelial Cell Growth Medium Supplement Mix and 1% penicillin/streptomycin (P/S) on gelatin-coated tissue plates or crosslinking gelatin-coated glass cover slips^61^. CCL-110 cells, HEK 293T, and HEK 293 cells were purchased from American Type Culture Collection and cultured in high glucose Dulbecco’s MEM (DMEM) supplemented with 10% FCS, 1% P/S and 1% sodium pyruvate (CCL-110 cells) or 1% Glutamine, respectively.

### Coatings of culture dishes with cross-linked gelatin

Culture dishes or glass bottom dishes were coated with 0.5% gelatin for 1 h at RT, and subsequently exposed to 2.5% glutaraldehyde/PBS for 10 min at RT. After the solution was removed, 70% ethanol/H2O was added for 30 min, and subsequently washing with PBS for 5 times. Active aldehyde groups were neutralised using 2 mM Glycine/PBS overnight. Prior to cell seeding culture dishes were washed 4 times with PBS.

### DNA constructs, viral transductions, and siRNA transfection

Recombinant lentiviral vectors carrying EGFP-p20, LifeAct-EGFP, VE-cadherin-mCherry and VE-cadherin-EGFP have previously been used^8^. β5-tubulin-EGFP^62^ was kindly provided by Beat A. Imhof and amplified using primes 5′-CTTGGGCTGCAGGTCGACTCTAGAGGATCCATGAGGGAAATCGTGCAC-3′ and 5′-TTGATTATCGATAAGCTTGATATCGAATTCTTACTTGTACAGCTCGTC-3′, subcloned into pFUGW vector using BamHI and EcoRI restriction sites. Raichu-Rac1^30^ was amplified with the primers 5′-TGCAGGTCGACTCTAGAGTGGCAAGAATTCGGCATG-3′ and 5′-TCGATAAGCTTGATATCGTTACAACAGCAGGCATTTTC-3′ and subcloned into a PFUGW vector using BamHI and EcoRI restriction sites.

### Lentivirus production

Lentivirus particles were generated in 293T cells cultured in DMEM (high glucose) medium in 15-cm cell culture dishes. Cells were transfected with the pFUGW vector carrying the gene of interest, the packaging vectors (pCMV-ΔR8.74), and the VSV glycoprotein-carrying vector (pMD2G,). In particular, the plasmids pFUGW-gene of interest (23 µg), pCMV-ΔR8.74 (23 µg), and pMD2G (11.5 µg)] were dissolved in 1.725 ml DMEM medium w/o FCS and antibiotics (DMEM−/−). The transfection solution, consisting of 124.2 µl of the transfection reagent PEI (1 mg ml^−1^) was dissolved in 1600.8 µl DMEM−/− and incubated at RT for 20 min; it was subsequently added to the plasmid mixture, and drop-wise added to the 293T cells. After 14–16 h of incubation, a medium exchange was performed using 20 ml fresh, pre-warmed full DMEM medium. After 24 h of culturing the medium was collected and cleared at 3000 r.p.m. for 10 min. The supernatant was filtered through 0.45-µm filters, and the virus was concentrated by ultracentrifugation (1.5 h at 25,000 r.p.m.; 4 °C). The pellet was resuspended in 150 µl PBS containing 1% BSA, and aliquots were stored at −80 °C until use.

### Adenovirus amplification

Adenovirus carrying VE-cadherin-EGFP, EGFP and N17Rac1, kindly provided by Masatoshi Takeichi^49^ and Beata Wojciak-Stothard^63^, respectively, were amplified by the following steps. HEK 293 cells were seeded in T75 cm^2^ tissue culture flasks and cultured for 24 h when reached 80–90% confluency. Medium was changed to DMEM containing w/o FCS and P/S supplemented with 1% Glutamine and 10 µl (15 MOL) of stock adenovirus. After incubation for 1 h, equivalent volumes of DMEM supplemented with 4% FCS, 1% Glutamine was added to reach a final concentration of 2% FCS in culture medium. After 2 additional days of culturing, four cycles of freeze-thaw-vortex was performed to release the virus. Depending on the amount of virus required, the virus yield can be significantly increased by two or more further culture cycles. For this purpose, the respective suspension is distributed to 5 times or 10 times greater HEK293 cell culture area (80–90% confluence), cultured for two days and processed by another four cycles of freeze-thaw-vortex. For virus purification, the cells were scrapped from the substrate and spinned down at 500 × *g* for 10 min, and the cell pellet was resuspended in 6 ml sterile PBS followed by another for four cycles of freeze-thaw-vortex. The lysate was cleared by 4000 x *g* at 4 °C for 20 min. For further purification 6 ml of the supernatant was mixed with 3,3 gr ultrapure CsCl, vortexed and separated at 1,76 × 10^5^ × *g* at 10 °C for 22 h. Afterwards, the virus fraction was collected with glycerol (10% final concentration), dialysed against BSS, aliquoted and stored at −80 °C until use.

### Lentivirus- and adenovirus-mediated gene transductions

HUVEC were transduced by lenti or adenoviruses by incubating the cells with viral particles suspended in endothelial growth medium w/o FCS and P/S but containing 3% poly-vinyl-pyrrolidone for 1 h to increase transduction efficiency. Cells were further cultured and subjected to the experiments after 24–72 h. Expression of the protein of interest was evaluated by immune fluorescence and Western blotting, respectively.

### siRNA-mediated downregulation of VEGFR2 and Nrp1 in HUVEC

The VEGFR2 and Nrp1 siRNAs were obtained as on-target plus smart pools from Dharmacon (Lafayette, CO, USA). Non-targeting siRNAs were purchased as on-target plus individual siRNAs from Dharmacon. A total of 25–100 nM of specific or control siRNA was introduced into the cells using the MATra-siRNA reagent (#PK-CT-2021-020) and a magnet plate (#PK-CT-2011-000) according to the manufacturer’s recommendations (Promokine, Heidelberg, Germany). Cells were used for experiments at 24–72 h after transfection.

### Western blot analysis

Cells were lysed in SDS sample buffer (0.18 M Tris, 6% SDS, 30% glycerine, 80 mg l^−1^ Bromophenol Blue, 10 mM DTT). The protein concentration in SDS containing sample buffer was determined by Amido-black protein assay^64^. In particular, equal amounts of protein were separated by 8–10% polyacrylamide gel electrophoresis, wet transferred to a PVDF membrane. The membrane was incubated with goat anti-VE-cadherin (1:500, #SC-6458, Santa Cruz Biotechnology, Heidelberg, Germany), rabbit anti-VEGFR2 (1:1000, #2479L, Cell Signalling Technology, Beverly, MA, USA), rabbit anti-Nrp1 (1:1000, #3725S, Cell Signalling Technology, Beverly, MA, USA), mouse anti-α-tubulin (1:5000, #T5168, Sigma, Munich, Germany), mouse anti-γ-catenin (1:1000, #610254, BD Biosciences, San Jose, CA, USA), mouse anti-N-cadherin (1:1000, #610920, BD Biosciences, Heidelberg, Germany), and mouse anti-EGFP (1:2500, #632569, Clontech, California, USA) at 4°C overnight, followed by incubation with IRDye 600 CW and 800 CW infra-red secondary anti-mouse, -goat, and -rabbit antibodies (1:10000, LI-COR Biotechnology, Bad Homburg, Germany). Immune reactive bands were detected using the LICOR Infra-red reading system according to the manufacturer’s instructions. Bands were semi-quantitatively analysed using Fiji image processing software. Uncropped scans of the blots are available in Supplementary Fig. 11.

### Staining of cell cultures and tissues

HUVECs were fixed in 2% PFA for 15 min at RT and washed three times for 5 min each with 1% BSA in PBS followed by mild permeabilization with 0.1% Triton X-100 (#28314, Thermo Fisher Scientific, Waltham, MA, USA) in PBS for 10 min at 4 °C. After three more washes with 1% BSA in PBS, cells were incubated with goat anti-VE-cadherin (1:50, #SC-6458, Santa Cruz Biotechnology, Heidelberg, Germany), rabbit anti-γ-catenin (1:50, #610254, BD Biosciences, Heidelberg, Germany), rabbit anti-EEA1 (1:200, #2411, Cell Signalling Technology, Beverly, MA), rabbit anti-Rab5 (1:1000, #ab18211, Abcam, Cambridge, UK), mouse anti-α-tubulin (1:1000, #T5168, sigma, Munich, Germany), rabbit anti-phospho myosin light chain II (ser19) (1:50, #3671, Cell Signalling Technology, Beverly, MA), and rabbit anti-VEGFR2 (1:200, #2479L, Cell Signalling Technology, Beverly, MA), overnight at 4 °C. Following 4 washes, the appropriate Alexa-Fluor-coupled secondary antibodies (1:200, Invitrogen, Darmstadt, Germany), in parallel with Phalloidin-TRITC (1:500, #P1951, Sigma, Munich, Germany), were added to the samples for 1 h at room temperature. Cells were rinsed, counterstained with DAPI (#28718-90-3, Sigma, Munich, Germany), and then mounted in DAKO fluorescent mounting medium (#S3023, Agilent Technologies, Böblingen, Germany). All animal experiments were carried out with the approval of the local animal ethics committees and performed with permission of the *Landesamt für Natur, Umwelt und Verbraucherschutz Nordrhein-Westfalen* and in accordance with guidelines for conducting animal experiments in a humane manner. The approval for mice experiments was granted to Max Planck Institute for Molecular Biomedicine (Laboratory of Ralf H. Adams), and the approval for tissue samples taken were granted to the Institute of Anatomy and Vascular Biology (Laboratory of Hans Schnittler).

For endothelial-specific loss-of-function of *Dll4*, we interbred *Pdgfb-iCre* ^T/+^ transgenic mice^65^ and *Dll4* ^loxlox^ mice^66^. Mice of both genders (1:1 ratio) were provided by the animal facility at the Max Planck Institute of Molecular Biomedicine (Münster, Germany). Pups obtained from mating of *Dll4* ^loxlox^ and *Pdgfb-iCre* ^T/+^ *Dll4* ^loxlox^ mice were injected from P1 to P3 with 50 µg tamoxifen and further analysed at P6. *Dll4* ^i∆EC^ mutant pups (Cre+) were compared to control (Cre-) littermates. For treatment of P5 C57BL/6 mice, provided by the animal facility at the Max Planck Institute of Molecular Biomedicine (Münster, Germany), of both genders (1:1 ratio) DAPT dissolved in 10% absolute ethanol and 90% peanut oil or vehicle was injected into the stomach of mice according to the body weight (0.1 mg kg^−1^ body weight), and this treatment was repeated after 12 h. Pups were analysed at 24 h from the first injection.

For ex vivo investigations, eyes of both genders (1:1 ratio) were collected from P7 Wistar rats provided by ZTE of University of Münster (Münster, Germany), from P6 wild-type (WT) mice, from P6 transgenic mice expressing LifeAct-EGFP in the retina vessels^17^ provided by the Max Planck Institute of Biochemistry, Department of Molecular Medicine (Munich, Germany), and from P6 *Dll4* ^i∆EC^ and littermate control mice. All eyes and E14.5 yolk sac and mature vena cava from WT mice were collected and fixed in 4% PFA for 2 h on ice. Samples were dissected, permeabilized and blocked in PBS containing 1% BSA and 0.3% Triton X-100 (blocking buffer) at RT for 2 h. Samples were then incubated with goat anti-VE-cadherin (1:50, #AF1002, R&D Systems, Wiesbaden-Nordenstadt, Germany) or rabbit anti-ERG (1:100, #ab110639, Abcam, Cambridge, UK), rabbit anti-phospho myosin light chain II (ser19) (1:50, #3671, Cell Signalling Technology, Beverly, MA) or rabbit anti-ARPC2 (1:200, #07-227, Merck Millipore, MA, USA) overnight at 4 °C. After four washing steps, samples were incubated with the corresponding Alexa-Fluor-coupled secondary antibody in blocking buffer for 2 h at RT. Samples were washed with washing buffer (0.5% BSA and 0.15% Triton x-100 in PBS) for 4 times and subsequently mounted in DAKO fluorescent mounting medium.

### Microscopy

For phase-contrast live-cell imaging, cells were placed on a gelatin-coated 12-well plate, and automatically imaged with an Axio observer Z1 (Carl Zeiss, Oberkochen, Germany) supplied with humidity and 5% CO_2_ using an LDA-plan 10× or 20× objective lens.

For fluorescence live-cell imaging, cells were seeded on crosslinking gelatin-coated custom-made 35 mm glass-bottomed dishes. Time-lapse recordings were performed using a spinning-disc confocal microscope (SDM), (Carl Zeiss, Oberkochen, Germany) equipped with definite focus and a heated stage that ensures humidity and 5% CO_2_. Live cells were imaged either using an Apo 1.3 oil 40× or alpha Plan Apo 1.46 oil 63× objective and the appropriate lasers for excitation and emission.

Structural illumination microscopy (SIM) was carried out using a Zeiss Elyra/LSM 780 microscope. The SIM images were acquired using a 100× or 63× plan-Apochromat oil-immersion objective and an electron-multiplying CCD camera with the use of 405, 488, 563, and 647 nm diode lasers. Images were reconstructed and processed for structure illumination with Zen imaging software (Carl Zeiss, Oberkochen, Germany). The alignment parameters for the different channels were obtained from measurements of a multispec calibration slide (170 nm multi-wavelength fluorescent beads, Carl Zeiss) taken with the same camera setup as the biological samples.

### Automated cell tracking using phase-contrast images

A novel software, the phase contrast based (PhaCoB) cell tracker,  was created to analyse morphodynamic parameters of phase-contrast time-lapse sequences. The automated cell tracking of phase-contrast images of EC cultures is based on a combination of two probabilistic models. Firstly, a Markov Random Field model for binary valued segmentations with higher-order Pn-Potts potentials^67,68^ is applied to each image of the sequence independently to provide foreground probabilities for each pixel in the image. These probabilities are then used to generate hypotheses for elliptically shaped foreground blobs that consist of pixels with sufficiently high foreground probability and cannot be enlarged without changing their geometry (up to scale). Note, that the generated ellipses are not considered to be final cell detections at this step of the approach but rather form a superset of the true cell ellipses, leaving the final decision to subsequent steps of the approach. Secondly, collections of interacting trajectories are modelled by which each trajectory is a temporally contiguous sequence of ellipses. Trajectories may start and end at any time. The model for a single trajectory is a Markov chain model incorporating a motion model, e.g., a “constant velocity” model, as well as the assumption of “continuous” changes of the cell shape. The interaction between the trajectories reflects the assumption that the cells in the confluent layer do not overlap each other. The resulting joint probabilistic model favours large and consistent collections of trajectories composed from the cell candidates found in the previous step of the approach. Algorithmically, this is achieved by first randomly generating such collections from the model. The final decision is obtained by Bayes risk minimisation for an appropriately chosen loss function. The second part of the model generalises a previously published simpler model for collections of point processes^69^. The number of cells visible in the frames of time lapse videos varies in time. One reason for this is that cells can leave or enter the field of view. Consequently, we report the average number of tracked cells in Figs 4l, n and 8f.

In an alternative method, for the images shown in Figs 2k, 5l, 6e and 8i, the cell border was first outlined manually using Fiji software and the elongation factor was defined by the cell aspect ratio where the length of the major axis is divided by the minor axis of the ellipse that best fits the object.

### Quantification of morphology and dynamics

Quantification of cell migration was performed by manual cell tracking using the Fiji ‘Manual Tracking’ plugin (https://imagej.nih.gov/ij/plugins/track/track.html) and the ‘Chemotaxis and Migration Tool’. Migration was analysed by comparing parameters, including i) accumulated distance (the total distance that the cell travelled) and ii) Euclidean distance (the straight-line distance between the start and end point of cell travel). For quantification of the HUVEC perimeter, images labelled with VE-cadherin were first segmented using our own lab-developed software CellBorderTracker (CBT)^12^, and then the perimeter was determined using Fiji. Relative VE-cadherin concentration (Rel-VEcad-C) in cell cultures was quantified using CBT software as described previously^12^. Rel-VEcad-C was defined by dividing the integrated VE-cadherin intensity of the region of interest (ROI) by the total cell length of the ROI. For quantification the cell perimeters in mouse retinal ECs, the Z-stack images of VE-cadherin labelling were assessed and the cell outline was drawn manually. For the analysis of VE-cadherin intensity in mouse retinal ECs, VE-cadherin immune-labelled single junctions were selected and subjected to a threshold. The relative VE-cadherin intensity at the junction was quantified by dividing the integrated VE-cadherin intensity by the junction length within the ROI.

### Fluorescence resonance energy transfer (FRET) analysis

FRET-based evaluation of Rac activity was carried out 2 days after viral transduction of the FRET sensor Raichu-Rac-1, HUVECs were stimulated with either 50 ng ml^−1^ VEGF or PBS for control. After 24 h YFP and CFP images were recorded by Zeiss Axioplan2 upright microscope (Oberkochen, Germany) and the FRET ratio of YFP/CFP images were analysed using Fiji software^29^.

### Real-time PCR analysis in cell culture and in vivo retina

Total RNA was purified from HUVEC lysates using the GeneMatrix Universal RNA Purification Kit (#E3598-02, EURx, Berlin, Germany) following the manufacturer’s instructions. Complementary cDNA was synthesised by using 500 ng of RNA with the Reverse Transcriptase Core Kit (#RT-RTCK-03, Eurogentec, Liege, Belgium) according to the supplier’s protocol. (RT-PCR) RT-PCR was carried out with the use of a SYBR Green PCR Master Mix (#07-KK4600-03, KAPA2G Fast Readymix with Dye, Peqlab, Erlangen, Germany) using the following primers: 5′-GAGGGTCTCTCTCTTCCTCTTGT-3′ and 5′-CTCCTCTGACTTCAACAGCGACA-3′ for GAPDH; 5′-TCCAACAGTGGGCAGCGAAG-3′ and 5′-CATAGTAGCCCGGAGGACAC-3′ for DLL4; 5′-GATAGCTCGCGGCATTCCAAG-3′ and 5′-CTCAGCGCAGCCGTCATCTG-3′ for Hes-1; 5′-GAGCCGCCGCCGCAGGAAG-3′ and 5′-CGTGAGCATCCAGCAGTGGTAGC-3′ for VE-cadherin. The relative quantification of the target gene was normalised to the GAPDH control and calculated using 2^−ΔΔCt^.

Prior to performing RNA extraction of retina ECs, FACS was applied to sort retina ECs. Briefly, freshly dissected retinas of *Dll4* ^i∆EC^ P6 pups and control littermates were lysed in a collagenase solution for 40 min at 37 °C. The homogenates were filtered through a 70 µm cell strainer(#352350, Falcon) and further resuspended in DMEM with 10% FCS. Filtrates were centrifuged for 5 min at 300 × g and cells were incubated with antibody solution (rat anti-CD31-FITC (#RM5201, Invitrogen, Darmstadt, Germany) and rat anti-CD45-PE (#553081, BD Pharmingen, San Jose, CA, USA) for 30 min on ice. After washing, retinal cells were resuspended in DMEM with 3% FCS and CD31 +/CD45- cells were sorted using FACS Canto flow cytometer.

RNA extraction from FACS sorted retinal ECs was performed using RNeasy Plus Micro Kit (#74034, Qiagen, Hilden, Germany). First-strand cDNA was synthesised using the iScript cDNA synthesis kit (#170-8890, Bio-Rad, Hercules, CA, USA). RT-PCR was performed using TaqMan Gene Expression Master Mix (#4369016, ThermoFisher Scientific, Waltham, MA, USA) along with Taqman assay for *Cdh5* (#Mm00486938_m1, ThermoFisher Scientific, Waltham, MA, USA) and for *ActB* (#4369016, ThermoFisher Scientific, Waltham, MA, USA), used to normalise gene expression.

### In vitro fibrin gel angiogenesis assay

Fibrin gel angiogenesis assays were carried out using HUVECs that were seeded onto Cytodex 3 micro-carrier beads (#C-3275, Sigma, Munich, Germany) at a concentration of ~400 cells per bead for 4 h and then allowed to adhere overnight. Following overnight culture, the coated beads were suspended in a 2 mg ml^−1^ fibrinogen solution (#F-8630, Sigma, Munich, Germany) containing 0.15 units ml^−1^ of aprotinin (#A-1153, Sigma, Munich, Germany) and 0.625 units ml^−1^ of thrombin (#T-4648, Sigma, Munich, Germany) and plated in custom-made Petri dishes with 1 cm^2^ glass bottoms. After the fibrinogen solidified, 2 ml of EGM-2 medium (#CC-3162, Lonza, Basel, Switzerland) containing 10 ng ml^−1^ of recombinant human VEGF was added. Finally, CCL-110 cells were seeded on top of the fibrin gel at a concentration of 1 × 10^4^ cells per well.

### Endothelial tube formation in Matrigel

A total of 70 µl of Matrigel basement membrane matrix (#356234, Corning, New York) was added to custom-made Petri dishes with 1 cm^2^ glass bottoms and placed in the incubator for 30 min at 37 °C to allow polymerisation. After that, HUVECs were seeded on top of the Matrigel matrix at a concentration of 3 × 10^4^ cells per cm^2^. Two hours after seeding, the indicated concentrations of the active ARP2/3 inhibitors CK666 (100 µM) or CK548 (50 µM), in parallel with their inactive controls CK689 (100 µM) and CK312 (50 µM), were applied to the cells.

### Impedance measurements

Impedance measurements were performed using HUVECs that were seeded in custom-made measuring impedance chambers and the measurements were initiated when cells reached confluence. The indicated concentrations of DAPT were added and the impedance was monitored at 10-min intervals. From the impedance spectroscopy data, the *trans*-endothelial electrical resistance (TER) was determined using TER analytical software (MOS-Technologies, Telgte, Germany) and was calculated as TER(t)/TER(t = 0), which is the TER value at a given time point (t) divided by the TER at the time point zero.

### Statistics

Statistical analyses were performed using GraphPad Prism 6 software (GraphPad Software, La Jolla, CA, USA). Comparisons between two groups were conducted using Student’s *t* test, and analyses among more than two groups were performed using one-way ANOVA followed by Tukey’s post hoc test. Two-way ANOVA was applied to compare the means of two independent variables, followed by Sidak post hoc test. Differences with *P* values <0.05 were considered significant. The data are presented as the mean ± SEM of at least three independent experiments.

### Data availability

All the data will be made available from the authors upon reasonable request.

## Electronic supplementary material

Supplementary Information

Description of Additional Supplementary Files

Supplementary Movie 1

Supplementary Movie 2

Supplementary Movie 3

Supplementary Movie 4

Supplementary Movie 5

Supplementary Movie 6

Supplementary Movie 7

Supplementary Movie 8

Supplementary Movie 9

Supplementary Movie 10

Supplementary Movie 11

Supplementary Movie 12

Supplementary Movie 13

Supplementary Movie 14

Supplementary Movie 15

Supplementary Movie 16

Supplementary Movie 17

Supplementary Movie 18

## References

[1] Gerhardt H (2003). VEGF guides angiogenic sprouting utilizing endothelial tip cell filopodia. J. Cell. Biol..

[2] Jakobsson L (2010). Endothelial cells dynamically compete for the tip cell position during angiogenic sprouting. Nat. Cell. Biol..

[3] Gaengel K (2012). The sphingosine-1-phosphate receptor S1PR1 restricts sprouting angiogenesis by regulating the interplay between VE-cadherin and VEGFR2. Dev. Cell.

[4] Bentley K (2014). The role of differential VE-cadherin dynamics in cell rearrangement during angiogenesis. Nat. Cell Biol..

[5] Yamamoto H (2015). Integrin beta1 controls VE-cadherin localization and blood vessel stability. Nat. Commun..

[6] Abraham S (2009). VE-Cadherin-mediated cell-cell interaction suppresses sprouting via signaling to MLC2 phosphorylation. Curr. Biol..

[7] Carmeliet P (1999). Targeted deficiency or cytosolic truncation of the VE-cadherin gene in mice impairs VEGF-mediated endothelial survival and angiogenesis. Cell.

[8] Abu Taha A, Taha M, Seebach J, Schnittler HJ (2014). ARP2/3-mediated junction-associated lamellipodia control VE-cadherin-based cell junction dynamics and maintain monolayer integrity. Mol. Biol. Cell..

[9] Rajput C (2013). Neural Wiskott-Aldrich syndrome protein (N-WASP)-mediated p120-catenin interaction with Arp2-Actin complex stabilizes endothelial adherens junctions. J. Biol. Chem..

[10] Krause M, Gautreau A (2014). Steering cell migration: lamellipodium dynamics and the regulation of directional persistence. Nat. Rev. Mol. Cell. Biol..

[11] Breslin JW, Zhang XE, Worthylake RA, Souza-Smith FM (2015). Involvement of local lamellipodia in endothelial barrier function. PLoS ONE.

[12] Seebach J (2015). The CellBorderTracker, a novel tool to quantitatively analyze spatiotemporal endothelial junction dynamics at the subcellular level. Histochem. Cell. Biol..

[13] Abella JV (2016). Isoform diversity in the Arp2/3 complex determines actin filament dynamics. Nat. Cell Biol..

[14] Hall A (2005). Rho GTPases and the control of cell behaviour. Biochem. Soc. Trans..

[15] Hayer A (2016). Engulfed cadherin fingers are polarized junctional structures between collectively migrating endothelial cells. Nat. Cell. Biol..

[16] Millan J (2010). Adherens junctions connect stress fibres between adjacent endothelial cells. BMC Biol..

[17] Fraccaroli A (2012). Visualization of endothelial actin cytoskeleton in the mouse retina. PLoS. One..

[18] Gelfand MV (2014). Neuropilin-1 functions as a VEGFR2 co-receptor to guide developmental angiogenesis independent of ligand binding. Elife.

[19] Shen Q, Rigor RR, Pivetti CD, Wu MH, Yuan SY (2010). Myosin light chain kinase in microvascular endothelial barrier function. Cardiovasc. Res..

[20] Schnittler HJ, Wilke A, Gress T, Suttorp N, Drenckhahn D (1990). Role of actin and myosin in the control of paracellular permeability in pig, rat and human vascular endothelium. J. Physiol..

[21] Li Z (2005). Regulation of PTEN by Rho small GTPases. Nat. Cell. Biol..

[22] Riento K, Ridley AJ (2003). Rocks: multifunctional kinases in cell behaviour. Nat. Rev. Mol. Cell. Biol..

[23] Etienne-Manneville S (2013). Microtubules in cell migration. Annu. Rev. Cell. Dev. Biol..

[24] Mayor R, Etienne-Manneville S (2016). The front and rear of collective cell migration. Nat. Rev. Mol. Cell. Biol..

[25] McCue S (2006). Shear stress regulates forward and reverse planar cell polarity of vascular endothelium in vivo and in vitro. Circ. Res..

[26] Drenckhahn D, Wagner J (1986). Stress fibers in the splenic sinus endothelium in situ: molecular structure, relationship to the extracellular matrix, and contractility. J. Cell. Biol..

[27] Garrett TA, Van Buul JD, Burridge K (2007). VEGF-induced Rac1 activation in endothelial cells is regulated by the guanine nucleotide exchange factor Vav2. Exp. Cell. Res..

[28] Zeng H, Zhao D, Mukhopadhyay D (2002). Flt-1-mediated downregulation of endothelial cell proliferation through pertussis toxin-sensitive G proteins, beta gamma subunits, small GTPase CDC42, and partly by Rac-1. J. Biol. Chem..

[29] Tarbashevich K, Reichman-Fried M, Grimaldi C, Raz E (2015). Chemokine-Dependent pH elevation at the cell front sustains polarity in directionally migrating zebrafish germ cells. Curr. Biol..

[30] Kardash E (2010). A role for Rho GTPases and cell-cell adhesion in single-cell motility in vivo. Nat. Cell. Biol..

[31] Nakatsu, M. N., Davis, J. & Hughes, C. C. Optimized fibrin gel bead assay for the study of angiogenesis. *J. Vis. Exp.* 186 (2007). 10.3791/186 PMC2570172 18978935

[32] Nehls V, Drenckhahn D (1995). A microcarrier-based cocultivation system for the investigation of factors and cells involved in angiogenesis in three-dimensional fibrin matrices in vitro. Histochem. Cell. Biol..

[33] Phng LK, Stanchi F, Gerhardt H (2013). Filopodia are dispensable for endothelial tip cell guidance. Development.

[34] Pelham RJ, Chang F (2002). Actin dynamics in the contractile ring during cytokinesis in fission yeast. Nature.

[35] Blanco R, Gerhardt H (2013). VEGF and Notch in tip and stalk cell selection. Cold Spring Harb. Perspect. Med.

[36] De Smet F, Segura I, De Bock K, Hohensinner PJ, Carmeliet P (2009). Mechanisms of vessel branching: filopodia on endothelial tip cells lead the way. Arterioscler. Thromb. Vasc. Biol..

[37] Hellstrom M (2007). Dll4 signalling through Notch1 regulates formation of tip cells during angiogenesis. Nature.

[38] Ehling M, Adams S, Benedito R, Adams RH (2013). Notch controls retinal blood vessel maturation and quiescence. Development.

[39] Sauteur L (2014). Cdh5/VE-cadherin promotes endothelial cell interface elongation via cortical actin polymerization during angiogenic sprouting. Cell Rep..

[40] Lampugnani MG (1995). The molecular organization of endothelial cell to cell junctions: differential association of plakoglobin, beta-catenin, and alpha- catenin with vascular endothelial cadherin (VE-cadherin). J. Cell. Biol..

[41] Benedito R (2012). Notch-dependent VEGFR3 upregulation allows angiogenesis without VEGF-VEGFR2 signalling. Nature.

[42] Tsuji-Tamura K, Ogawa M (2016). Inhibition of the PI3K-Akt and mTORC1 signaling pathways promotes the elongation of vascular endothelial cells. J. Cell. Sci..

[43] Seebach J (2007). Regulation of endothelial barrier function during flow-induced conversion to an arterial phenotype. Cardiovasc. Res..

[44] Wang Y (2010). Moesin1 and Ve-cadherin are required in endothelial cells during in vivo tubulogenesis. Development.

[45] Sugden WW (2017). Endoglin controls blood vessel diameter through endothelial cell shape changes in response to haemodynamic cues. Nat. Cell. Biol..

[46] Hasan SS (2017). Endothelial Notch signalling limits angiogenesis via control of artery formation. Nat. Cell. Biol..

[47] Lee CC (2013). Disrupting the CXCL12/CXCR4 axis disturbs the characteristics of glioblastoma stem-like cells of rat RG2 glioblastoma. Cancer Cell. Int..

[48] Gebala V, Collins R, Geudens I, Phng LK, Gerhardt H (2016). Blood flow drives lumen formation by inverse membrane blebbing during angiogenesis in vivo. Nat. Cell Biol..

[49] Kametani Y, Takeichi M (2007). Basal-to-apical cadherin flow at cell junctions. Nat. Cell Biol..

[50] Levayer R, Lecuit T (2013). Oscillation and polarity of E-cadherin asymmetries control actomyosin flow patterns during morphogenesis. Dev. Cell.

[51] Kage F (2017). FMNL formins boost lamellipodial force generation. Nat. Commun..

[52] Bentley K, Mariggi G, Gerhardt H, Bates PA (2009). Tipping the balance: robustness of tip cell selection, migration and fusion in angiogenesis. PLoS Comput. Biol..

[53] Nakayama M (2013). Spatial regulation of VEGF receptor endocytosis in angiogenesis. Nat. Cell Biol..

[54] Gavard J, Gutkind JS (2006). VEGF controls endothelial-cell permeability by promoting the beta-arrestin-dependent endocytosis of VE-cadherin. Nat. Cell Biol..

[55] Wright TJ, Leach L, Shaw PE, Jones P (2002). Dynamics of vascular endothelial-cadherin and beta-catenin localization by vascular endothelial growth factor-induced angiogenesis in human umbilical vein cells. Exp. Cell. Res..

[56] Luxton GW, Gundersen GG (2011). Orientation and function of the nuclear-centrosomal axis during cell migration. Curr. Opin. Cell. Biol..

[57] Waterman-Storer CM, Worthylake RA, Liu BP, Burridge K, Salmon ED (1999). Microtubule growth activates Rac1 to promote lamellipodial protrusion in fibroblasts. Nat. Cell Biol..

[58] Fraccaroli A (2015). Endothelial alpha-parvin controls integrity of developing vasculature and is required for maintenance of cell-cell junctions. Circ. Res..

[59] Nohata N (2016). Temporal-specific roles of Rac1 during vascular development and retinal angiogenesis. Dev. Biol..

[60] Hoelzle MK, Svitkina T (2012). The cytoskeletal mechanisms of cell-cell junction formation in endothelial cells. Mol. Biol. Cell..

[61] Kronstein R (2012). Caveolin-1 opens endothelial cell junctions by targeting catenins. Cardiovasc. Res..

[62] Ballestrem C, Wehrle-Haller B, Hinz B, Imhof BA (2000). Actin-dependent lamellipodia formation and microtubule-dependent tail retraction control-directed cell migration. Mol. Biol. Cell.

[63] Wojciak-Stothard B, Ridley AJ (2003). Shear stress-induced endothelial cell polarization is mediated by Rho and Rac but not Cdc42 or PI 3-kinases. J. Cell Biol..

[64] Dieckmann-Schuppert A, Schnittler HJ (1997). A simple assay for quantification of protein in tissue sections, cell cultures, and cell homogenates, and of protein immobilized on solid surfaces. Cell Tissue Res..

[65] Claxton S (2008). Efficient, inducible Cre-recombinase activation in vascular endothelium. Genesis.

[66] Koch U (2008). Delta-like 4 is the essential, nonredundant ligand for Notch1 during thymic T cell lineage commitment. J. Exp. Med..

[67] Kohli, P., Kumar, P. & Torr, P. *P3 and Beyond: Solving Energies with Higher Order Cliques. IEEE Computer Society Conference on Computer Vision and Pattern Recognition (CVPR)* 1–8 (IEEE, Los Alamitos, USA, 2007).

[68] Zhang, J., Djolonga, J. & Krause, A. *International Conference on Computer Vision* (ICCV, Los Alamitos, USA, 2015).

[69] Sixta, T. & Flach, B. *19th International Conference on Medical Image Computing and Computer Assisted Intervention* (Springer International Publishing AG, Cham, Switzerland, 2016).

