# Supplementary material for: Polarized actin and VE-cadherin dynamics regulate junctional remodelling and cell migration during sprouting angiogenesis
Source: Nat Commun. 2017 Dec 20;8:2210. doi: 10.1038/s41467-017-02373-8 (PMC5738342; doi:10.1038/s41467-017-02373-8)
Supplement: Supplementary file 1 — Supplementary Information [file 41467_2017_2373_MOESM1_ESM.pdf]

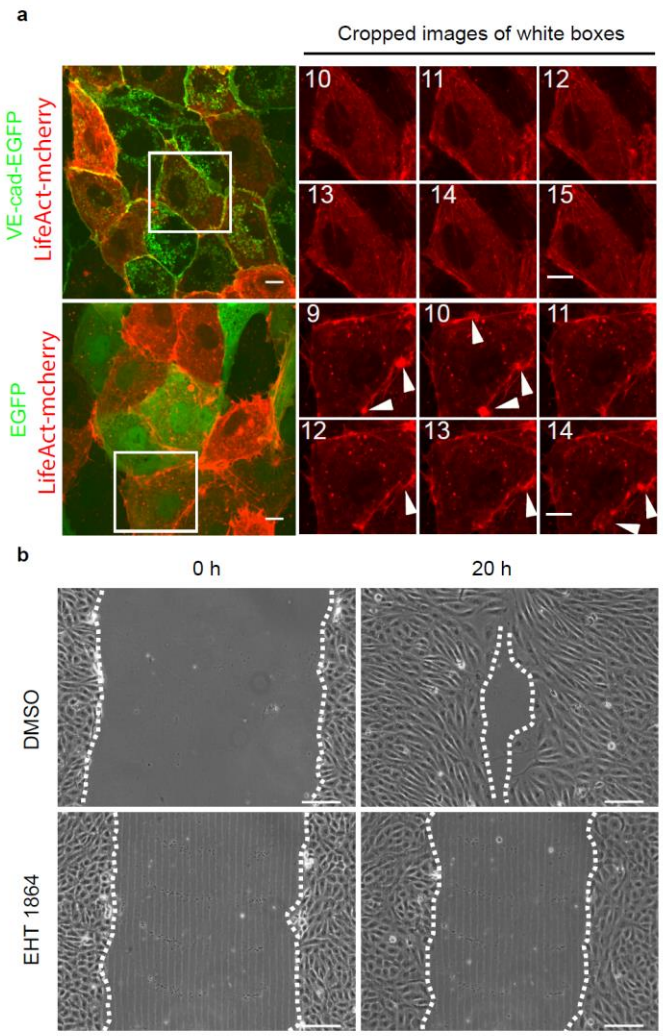


**Supplementary Figure 1**. Rac inhibition by EHT1864 blocks directed cell migration in scratch assay. **a** Time lapse recordings of LifeAct-mcherry in VE-cadherin-EGFP or EGFP overexpressing HUVEC. Note, JAIL formation (arrowheads) was blocked by VE-cadherin overexpression. Time scale: min; scale bar: 10 µm. **b** Confluent HUVEC were scratched under control conditions (DMSO, upper panel) or after 10 µM EHT1864 (lower panel). Representative images were taken at the time points as indicated. Dotted line indicates the border of migrating front. Results refer to Figure 2n, o. Scale bar: 200 µm.

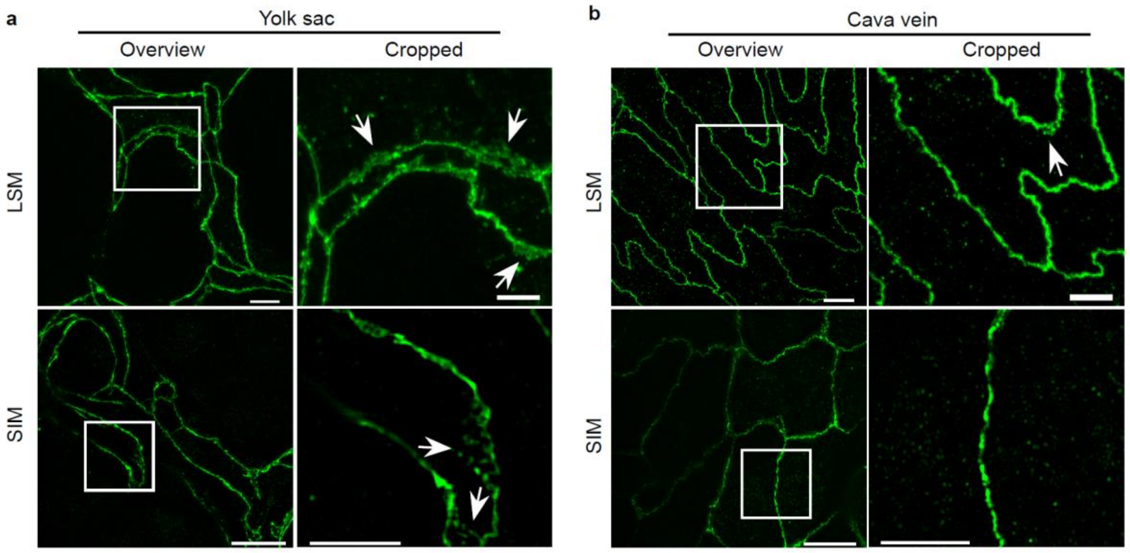


**Supplementary Figure 2**. JAIL formation in developing and mature vessels in vivo. **a** Yolk sac from mouse E14.5 embryos and **b** adult cava vein ECs were immune stained with VE-cadherin antibody and examined by maximal intensity projection of confocal Z-stacks using LSM (upper panels) and by one z-layer using SIM (lower panels). High resolution images of the yolk sac uncovered VE-cadherin clusters (arrows) forming large plaques in the yolk sac while the junctions of adult cava vein displayed an overall linear VE-cadherin appearance. Note: only a subset of the VE-cadherin plaques are visible in one z-plane by SIM. Scale bar in the overview images is 10 µm, and in the cropped images is 5 µm.

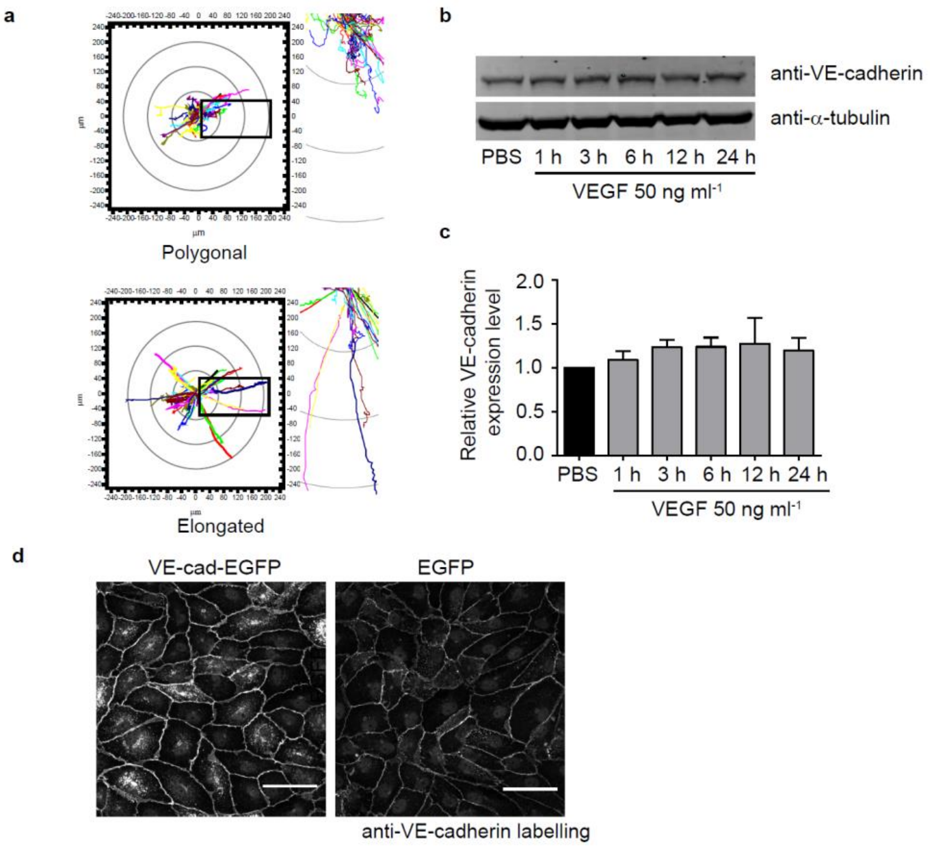


**Supplementary Figure 3**. VEGFR2 controls VEGF induced EC elongation in synergism with Nrp1. **a** Confluent HUVECs were treated with 50ng ml$^{-1}$ VEGF. Tracking plots illustrate directed cell migration of VEGF-induced elongated cells, whereas random movement was documented for polygonal cells even in the presence of VEGF (Refer to Figure 4d). **b** Confluent HUVEC cultures were treated with PBS or VEGF (50 ng ml$^{-1}$) as indicated followed by Western blot analysis. α-tubulin served as an internal loading control. **c** Quantitative determination of VE-cadherin levels displayed a slight tendency towards a transient increase in VE-cadherin levels, but this remained non-significant determined by one-way ANOVA. Error bars represent ± SEM. **d** LSM of VE-cadherin immune-labelled cells after VE-cadherin-EGFP overexpression or EGFP expression. Results of **d** refer to Figure 4n. Data shown are representative of three independent experiments. Scale bar: 50 μm.

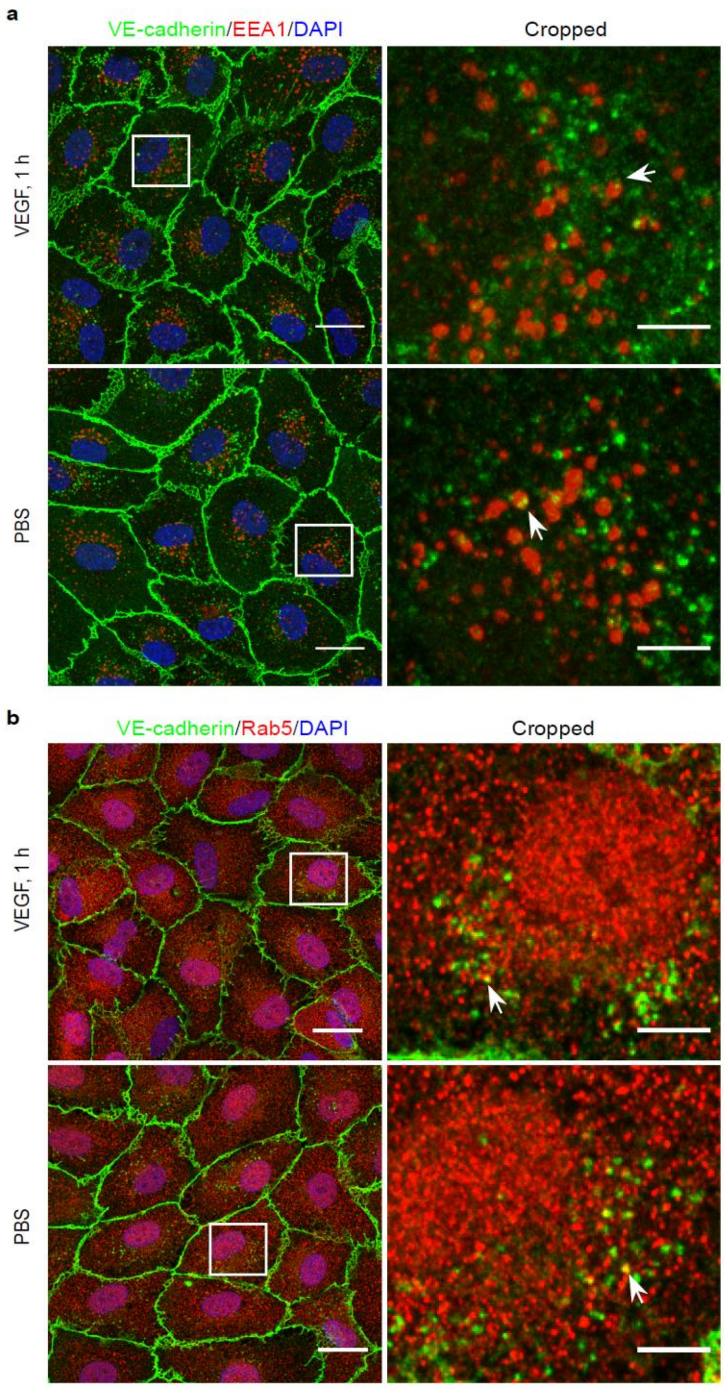


**Supplementary Figure 4**. Detection of VE-cadherin endocytosis in response to VEGF. Confluent HUVEC treated with 50 ng ml$^{-1}$ VEGF for 1 h were fixed and immune labelled with VE-cadherin, and (**a**) EEA1, and (**b**) Rab5. Few colocalization of VE-cadherin with (**a**) EEA1 and (**b**) Rab5 (indicated by arrows) was found both in PBS and VEGF treated cultures. Scale bars are 10 μm and 5 μm in the left panels and right panels, respectively.

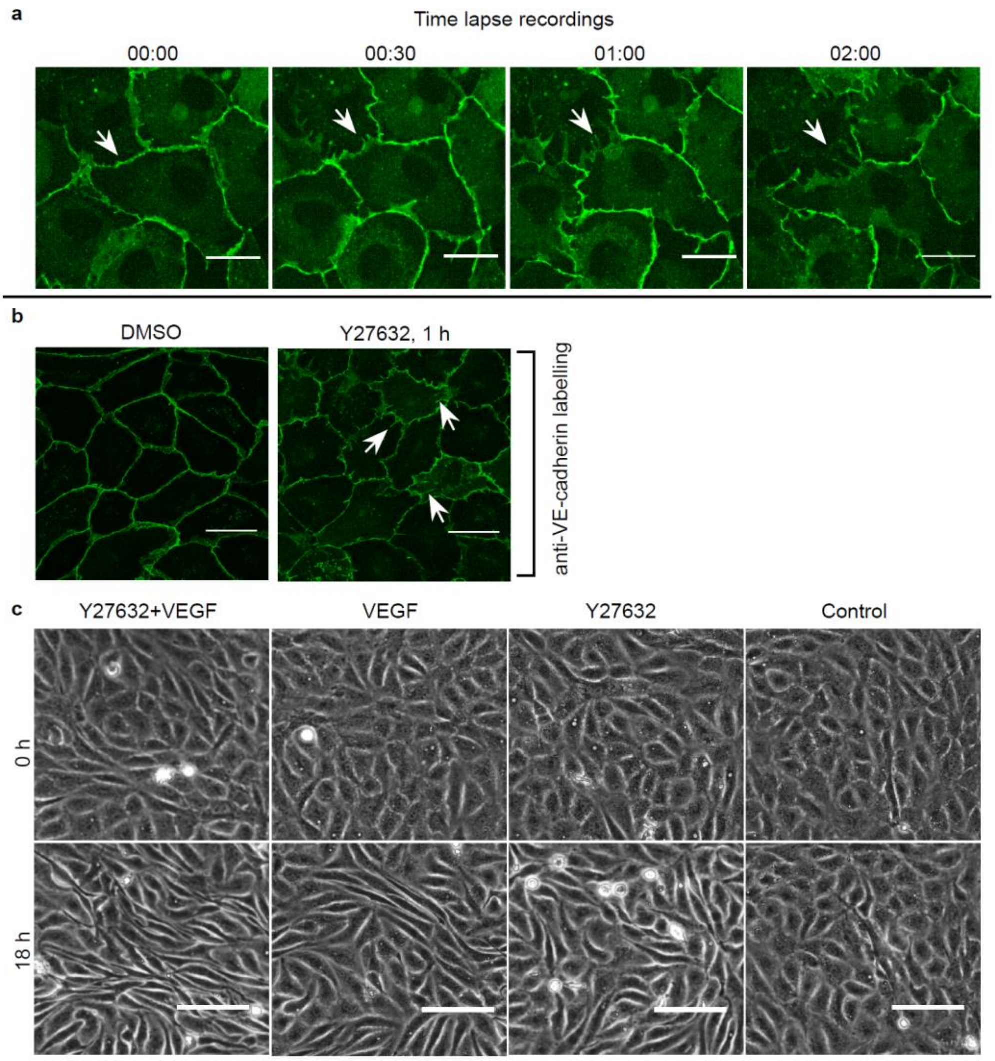


**Supplementary Figure 5**. Rock inhibitor Y27632 increases VE-cadherin invagination and cell elongation. **a** Time lapse images of VE-cadherin-EGFP expressing HUVEC treated by 10 µM Y27632 over time. Y27632 application induced VE-cadherin invagination formation (arrows). Time scale: hh:mm. Figure refers to supplementary Movie 9. Scale bar: 20 µm. **b** Confluent HUVECs treated with 10 µM Y27632 for 1 h were fixed and immune labelled with VE-cadherin. Arrows point development of VE-cadherin invagination. This result refers to Figure 5g-j. Scale bar: 30 µm. **c** Confluent HUVECs were pre-treated with the ROCK inhibitor Y27632 for 2 h followed by VEGF application for another 18 h. Both VEGF and Y27632 treatment induced HUVEC elongation, while Y27632 together with VEGF stimulation induced a syngeristic effect. This data refer to Figure 5k, l. Scale bar: 100 µm. Data shown are representative of three independent experiments.

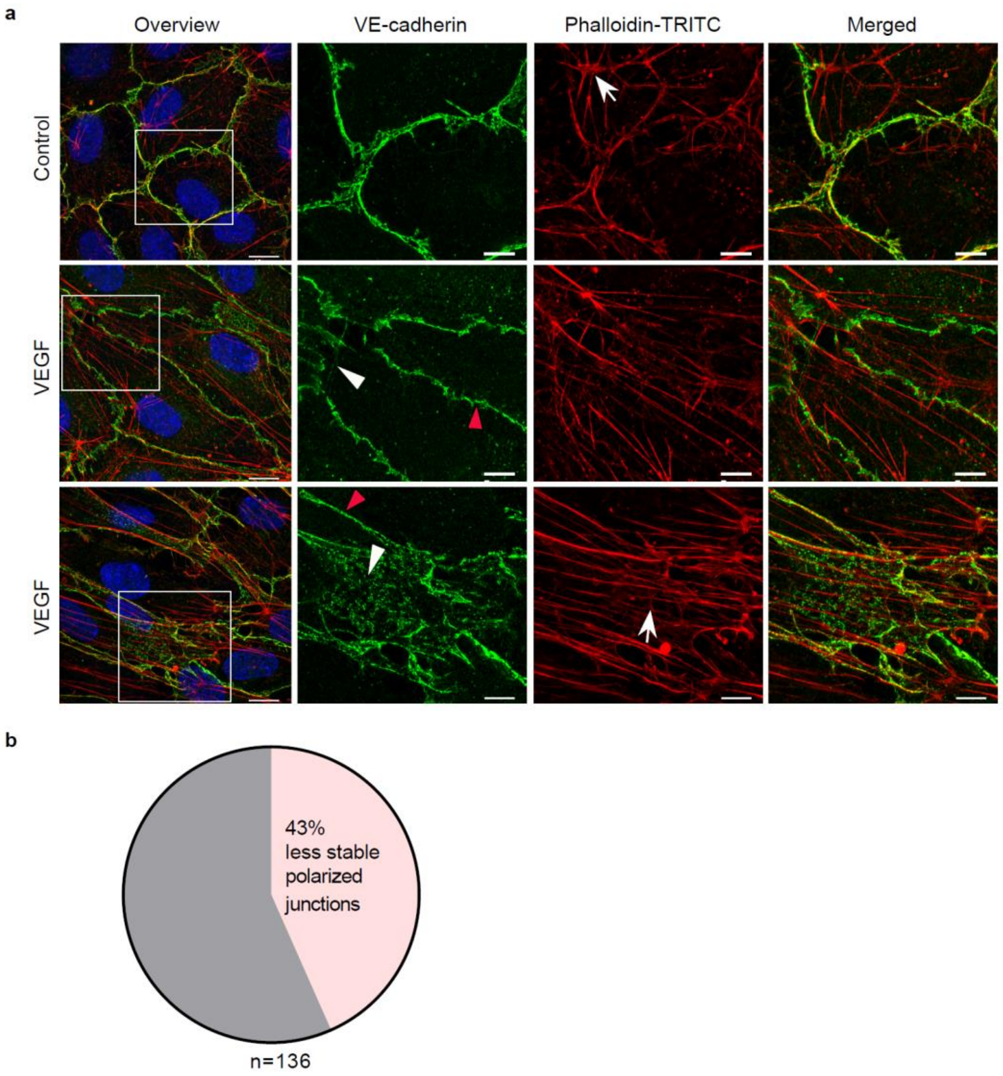


**Supplementary Figure 6**. SIM of VE-cadherin and actin in VEGF treated HUVEC culture. **a** Confluent HUVEC cultures were treated by either PBS (control) or VEGF for 24 h, subsequently fixed and labelled by VE-cadherin antibody and phalloidin-TRITC for actin filament staining. (upper panel) Under control conditions VE-cadherin and actin filaments localizes at cell junctions. A few stress fibres are visible (arrow). (middle panel) After VEGF treatment an interrupted VE-cadherin pattern was visible at the cell poles (white arrowhead) while lateral junctions displayed an overall linear VE-cadherin pattern (red arrowheads). (lower panel) VEGF treatment also caused the formation of large VE-cadherin adhesion plaques (white arrowhead) as a result of transient JAIL formation. Stress fibers are indicated (arrow). Scale bars indicate 10 μm in the overview images and 5 μm in the cropped images. **b** The Pie graph shows the percentage of interrupted VE-cadherin patterning at cell poles in elongated cells. Data shown are representative of three independent experiments.

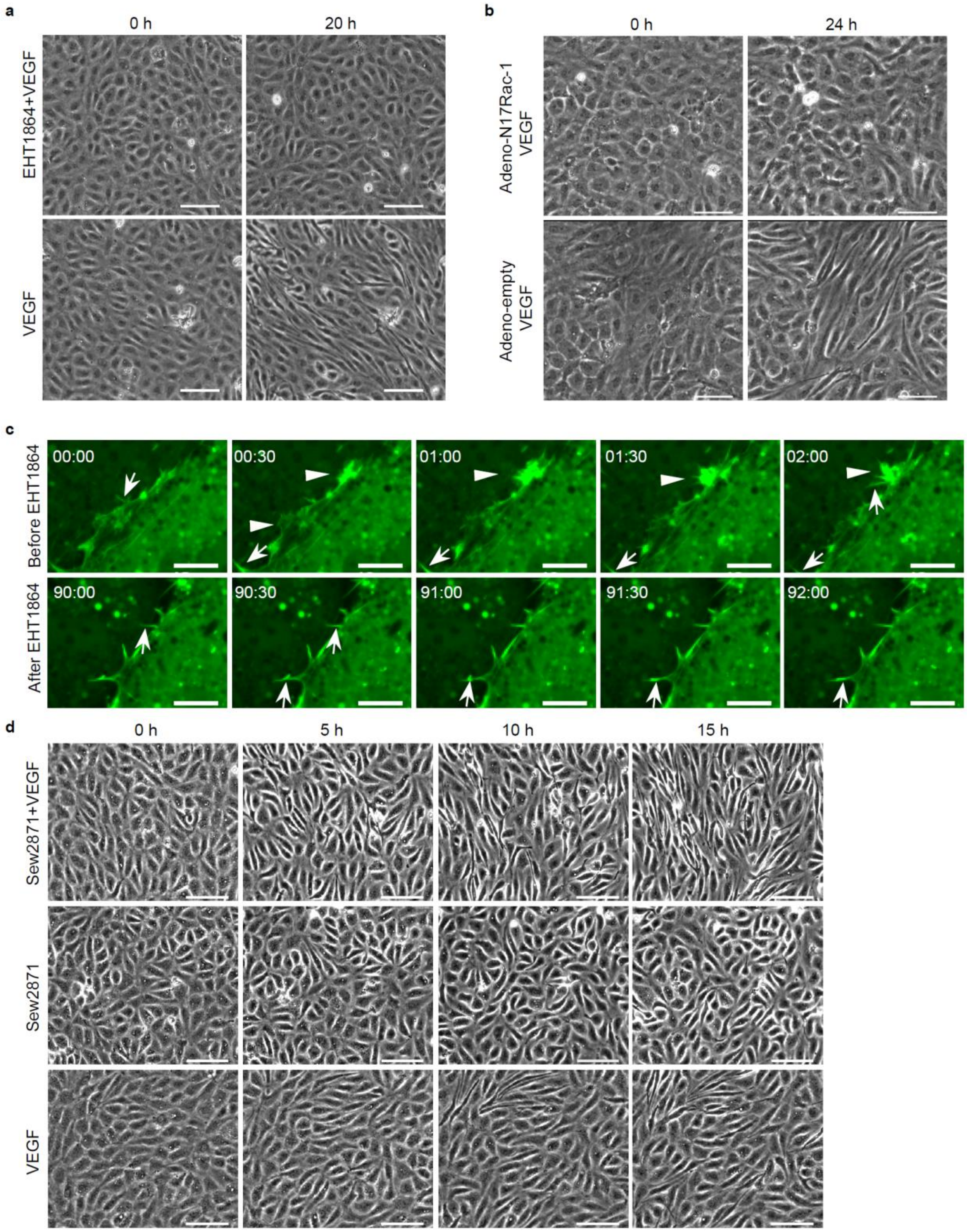


**Supplementary Figure 7**. Rac activation is required for VEGF induced cell elongation. **a** Application of the Rac inhibitor EHT 1864 (10 µM) blocked VEGF induced cell elongation (upper panel) in comparison with VEGF only (lower panel). Compare Supplementary Movie 14. **b** Confluent HUVEC cultures expressing Adeno-N17Rac-1 totally blocked VEGF induced cell elongation (upper panel), while transduction with an empty adenovirus vector for control had no effect (lower panel). **c** Time lapse recordings of LifeAct-EGFP dynamics in HUVECs under control conditions and after EHT1864 treatment. (upper panel) Under control conditions the characteristic JAIL formation (arrowheads) is visible with filopodia-like structures (arrows)

appearing at cell junctions are detected (lower panel) 90 min after EHT1864 application JAIL formation was abolished while filopodia like structures still appeared (arrows). The time format is mm:ss, compare Supplementary movie 14. **d** Confluent HUVEC pretreated with Sphingosine1 phosphate receptor agonist Sew2871 (10 µM ) were subsequently stimulated with VEGF. Images taken at time points as indicated demonstrate the morphological changes. These results refer to Figure 8i. Scale bars in the **a**, **b**, and **d** are 100 µm; scale bars in the **c** are 10 µm.

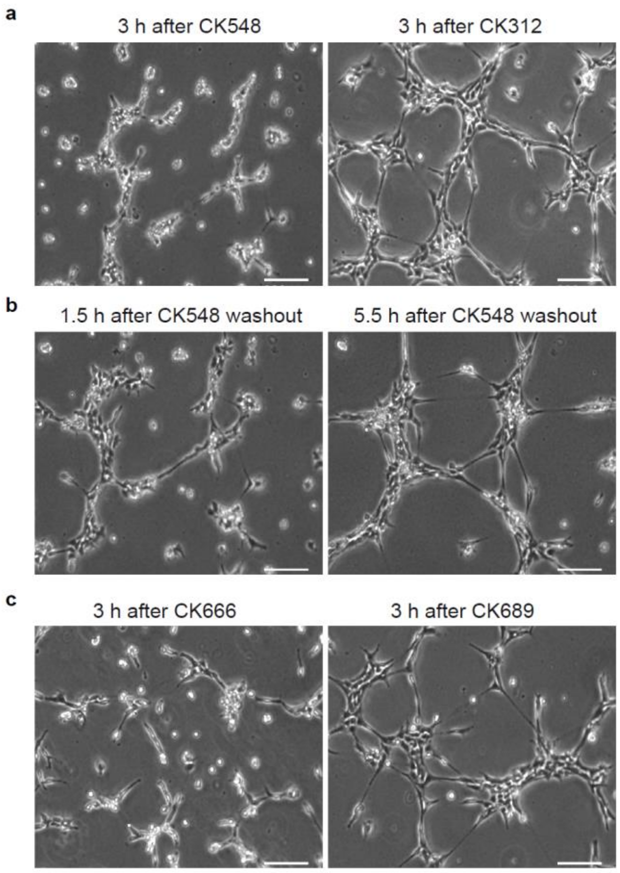


**Supplementary Figure 8**. ARP2/3 complex inhibitors block tube formation in Matrilgel assay. HUVECs were seeded on top of a polymerized Matrigel basement membrane matrix. **a** Two hours after cell seeding 50 µM of the CK548 or its inactive control CK312 respectively were added. Images were taken 3 h after inhibitor administration. **b** Three hours after CK548 treatment, the medium was washed out and replaced with fresh complete medium containing no inhibitors. Images were taken 1.5 h and 5.5 h after washing. **c** Alternatively, 100 µM of the ARP2/3 complex inhibitor CK666 or its inactive control CK689 were applied to the culture. Images were taken 3 h after inhibitor administration. Scale bar: 200 µm. Data shown are representative of three independent experiments.

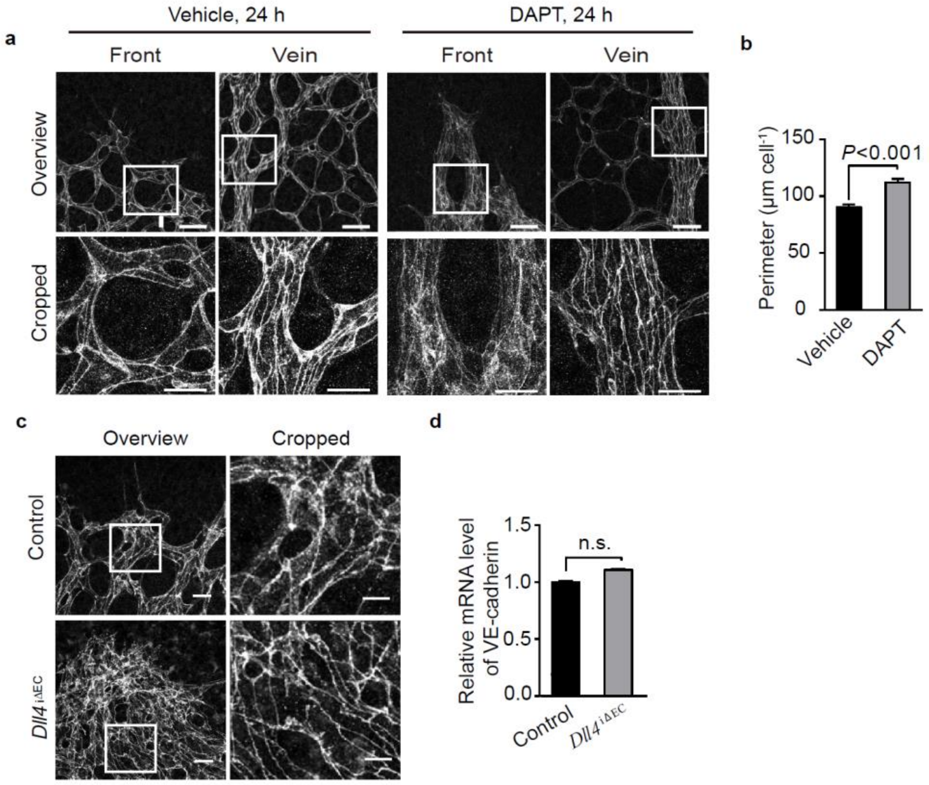


**Supplementary Figure 9.** Inhibition of Notch signalling induces EC elongation. **a, b** Wild-type P6 mice were treated with DAPT or vehicle, as a control, for 24 h. Retinas were dissected and stained for VE-cadherin, and this labelling was used to conduct a manual quantitative determination of the cell junction perimeter. **a** Comparison of VE-cadherin labelling of vehicle (upper panel) or DAPT-treated mice (lower panel) revealed a characteristic increase in sprouting angiogenesis in DAPT-treated animals. Scale bar in the overview and cropped images is 40 μm and 20 μm respectively. **b** Quantification of the perimeter of vein endothelium in DAPT-treated mice also displayed a significant increase; n=43 and 50 cells for vehicle and DAPT treatment, respectively. **c** Whole-mounted P6 retinas of control and *DLL4* $^{i\Delta EC}$ mice were labelled with VE-cadherin. Representative images of VE-cadherin staining demonstrate an increase in sprouting angiogenesis. Scale bar in the overview and cropped images is 50 μm and 10 μm respectively. **d** Analysis of VE-cadherin mRNA level in the sorted retinal ECs of control and *DLL4* $^{i\Delta EC}$ mice displays an unchanged mRNA level. Significance difference was determined by unpaired student's t-test. Error bars represent ± SEM.

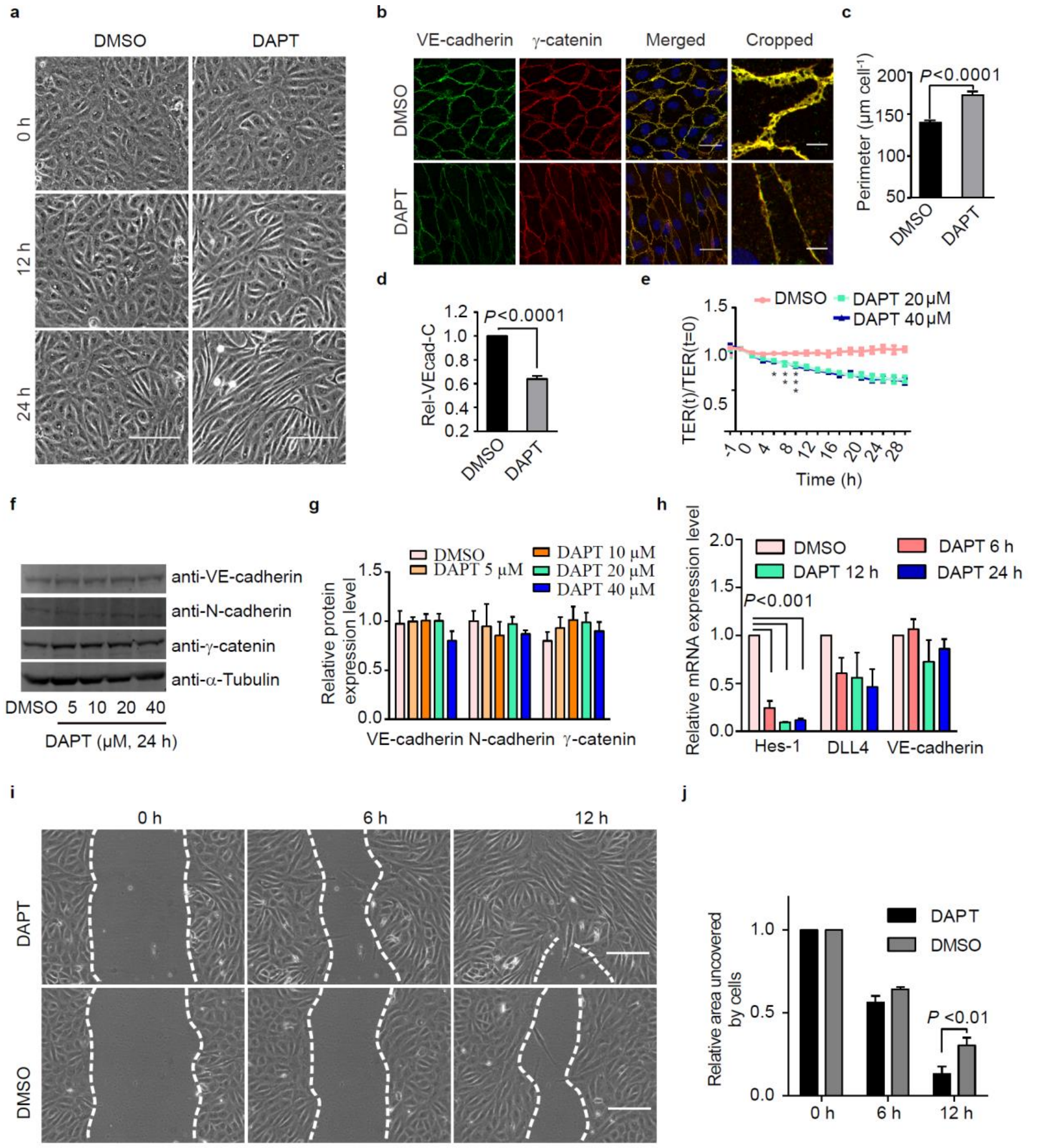


**Supplementary Figure 10.** γ-secretase inhibition by DAPT increases cell elongation and migration. **a** Time lapse recording of confluent HUVEC cultures with 20 µM DAPT treatment or DMSO as a control over 24 h. DAPT progressively induced cell elongation. Scale bar: 150 µm. **b** After 24 h of treatment cultures were immune labelled for VE-cadherin and γ-catenin. Scale bars in the overview and cropped images are 30 µm and 5 µm respectively. Quantification of (**c**) cell perimeter and (**d**) Rel-VEcad-C using CBT. n=60 cells. **e** TER plot of HUVEC cultures as a function of time. DMSO or DAPT were added at time point zero. p-values are indicated by *; * = p< 0.05; ** = p< 0.001; *** = p< 0.0001; n.s. = not significant. **f, g** Quantitative western blot analyses of VE-cadherin, N-cadherin, and γ-catenin in DAPT- and DMSO-treated HUVEC cultures (n=3). α-tubulin served as an internal control. **h** DAPT treated HUVEC cultures were probed for Hes-1, DLL4 and VE-cadherin mRNA levels at the indicated time points (n=3). Gene expression levels

were normalized to GAPDH. **i, j** Scratch assay of HUVEC cultures in the presence and absence of DAPT. Prior to scratching, the confluent cultures were pre-treated with 20 µM DAPT for 16 h, which caused increased cell elongation while control DMSO treated cells remained polygonal. Subsequently, cultures were scratched using a 200µl pipette tip and fresh medium containing 20 µM DAPT was substituted. **i** Phase contrast images at various times after scratch are shown, as indicated. Note that wound closure is accelerated when cells reached an elongated cell shape prior to scratching, as is the case during DAPT treatment. Scale bar: 150 mm. **j** Comparison of the area that was not uncovered by cells over time during the wound closure. n=6 movies pooled from two independent experiments. Significance difference was determined by unpaired student's t-test for experiment **c**, **d** and two-way ANOVA for experiment **e**, **h**, **j**. Error bar represent ± SEM.

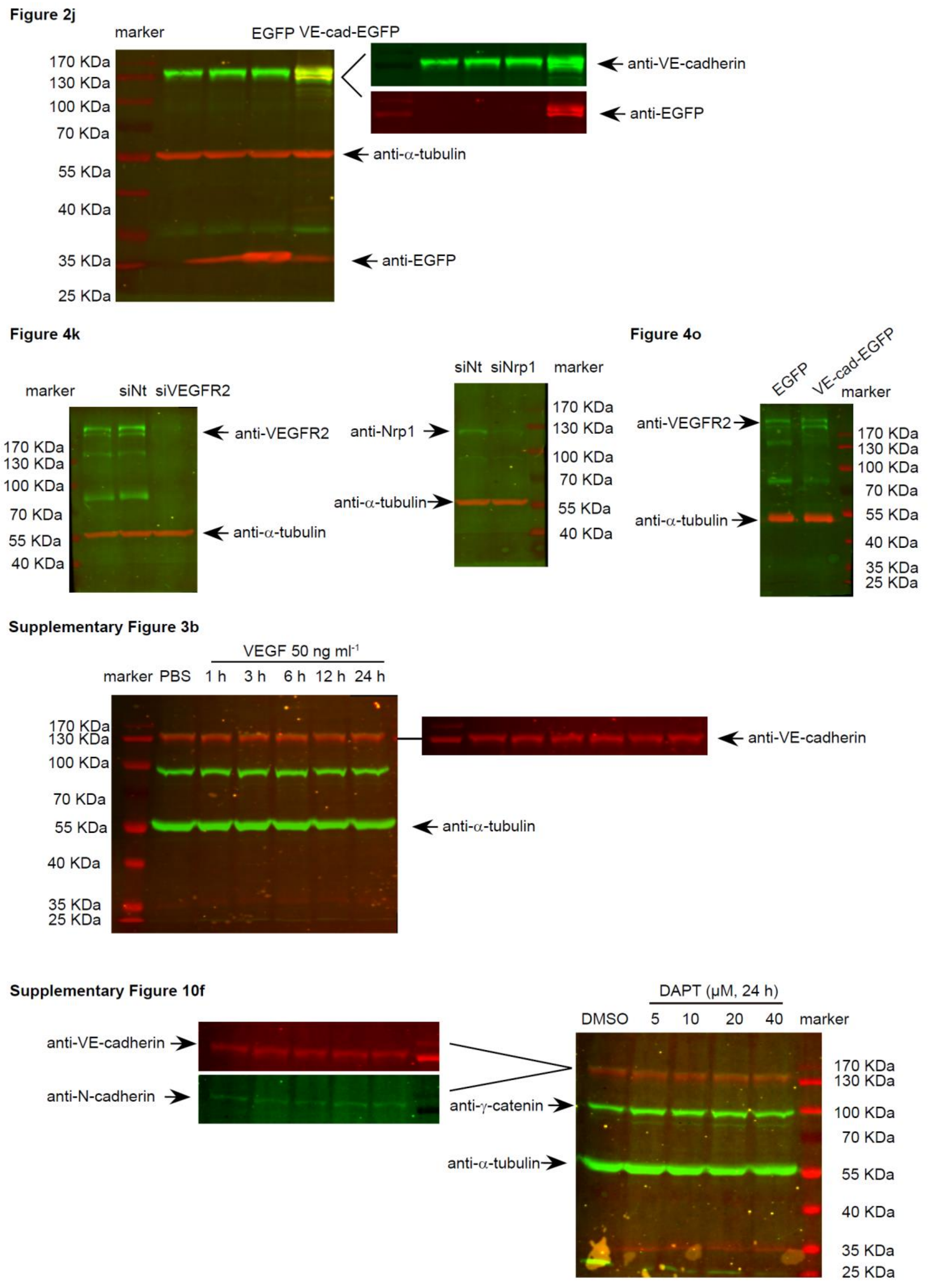


**Supplementary Figure 11**. Uncropped Western blots relate to Figure 2j, 4k, 4o, and Supplementary Figure 3b, 10f.
