# Supplementary material for: Polarized actin and VE-cadherin dynamics regulate junctional remodelling and cell migration during sprouting angiogenesis
Source: Nat Commun. 2017 Dec 20;8:2210. doi: 10.1038/s41467-017-02373-8 (PMC5738342; doi:10.1038/s41467-017-02373-8)
Supplement: Supplementary file 2 — Description of Additional Supplementary Files [file 41467_2017_2373_MOESM2_ESM.pdf]

Description of Additional Supplementary Files

File Name: Supplementary Movie 1
Description: Time lapse recordings of HUVEC during sheet migration investigated in a scratch assay. Confluent HUVEC were scraped using a 200 µl pipette tip and recorded by phase contrast microscope. The leader cells developed lamellipodia and follower cells subsequently elongate and increase migration. The video is verbally annotated.

File Name: Supplementary Movie 2
Description: Dynamics VE-cadherin-EGFP under endothelial wound healing conditions (scratch assay). Confluent HUVEC cultures expressing VE-cadherin-EGFP were scraped followed by time lapse recording using spinning disc microscopy. Cells gradually increased elongation and the relative VE-cadherin concentration at the junctions diluted. Large VE-cadherin plaques appeared at the cell pole were clustered and incorporated into the junction forming liner or interrupted VE-cadherin patterning. Faint linear VE-cadherin with occasional small VE-cadherin plaques was observed in the lateral junctions. The video is verbally annotated.

File Name: Supplementary Movie 3
Description: Dynamics of VE-cadherin-mcherry and EGFP-p20 during cell migration in a scratch assay. Confluent HUVEC expressing both VE-cadherin-mcherry and EGFP-p20 were scraped and the time lapse recordings were performed by spinning disc microscopy.

File Name: Supplementary Movie 4
Description: Animation of JAIL dynamics. When relative VE-cadherin concentration is low, ARP2/3 complex (blue) dependent actin network formation (green) leads to JAIL formation that overlap the plasma membrane of neighbouring cells. At this site VE-cadherin molecules (white), freely floating in the plasma membrane, form adhesion dimers/multimers that cluster after the actin and ARP2/3 complex is dissembled and incorporate into the junctions, forming new VE-cadherin adhesion sites. The video is verbally annotated.

File Name: Supplementary Movie 5
Description: Application of VEGF induces endothelial cell elongation that forces directed cell migration. Phase Contrast microscopy of confluent HUVEC cultures treated with 50 ng ml^{-1} VEGF or PBS for control. VEGF stimulation gradually and partially induces cell elongation and cell migration. The video is verbally annotated.

File Name: Supplementary Movie 6
Description: VEGFR2 controls VEGF-induced cell elongation in synergism with Nrp1. HUVEC cultures were transfected with siVEGFR2, siNrp1 or siNt and subsequently exposed to VEGF. Phase Contrast microscopy was performed after VEGF application. siVEGFR2 totoally blocked VEGF induced cell elongation while siNrp1 moderately decreased VEGF induced cell elongation. The video is verbally annotated.

File Name: Supplementary Movie 7
Description: VE-cadherin-EGFP overexpression in HUVEC cultures blocks VEGF-induced cell elongation. Confluent HUVEC were transduced with high titer of VE-cadherin-EGFP adenovirus that leads to over-expression of VE-cadherin-EGFP within hours. Phase-Contrast microscopy was performed after VEGF application. VE-cadherin overexpression blocked VEGF induced cell elongation and cell migration. The video is verbally annotated.

File Name: Supplementary Movie 8
Description: VEGF-induced VE-cadherin dynamics and remodeling. Confluent HUVECs expressing EGFP-p20 were applied with VEGF. Quickly after VEGF application, VE-cadherin invaginations (white arrows) accompanied with occasional events of VE-cadherin endocytosis (red arrows) were observed. Furthermore, large VE-cadherin plaques (white dotted line) occurred after VEGF suggest VEGF application increased VE-cadherin dynamics. The video is verbally annotated.

File Name: Supplementary Movie 9
Description: Loss of tension due to ROCK inhibition dramatically increased VE-cadherin dynamics. Confluent HUVEC cultures expressing VE-cadherin-EGFP were stimulated with ROCK inhibitor Y27632. Y27632 application induced curved cell shape accompanied with increased VE-cadherin invaginations (red arrows). The video is verbally annotated.

File Name: Supplementary Movie 10
Description: VEGF triggers polarized microtubules (MTs) dynamics that induces and maintains cell elongation and migration. Confluent HUVEC cultures expressing β5tubulin-EGFP were applied with VEGF. VEGF increased MTs dynamics that leads to cell elongation at that site of the cells where the MTOC is located. 24 h after VEGF application, nocodazole was additionally added to the cell culture and nocodazole depolymerized the MTs, induced the roundness of the cell, and blocked cell migration. The video is verbally annotated.

File Name: Supplementary Movie 11
Description: Actin dynamics in HUVECs upon VEGF application. HUVEC cultures expressing LifeAct-EGFP were treated with VEGF followed by time lapse recording using spinning disc microscopy. The video is verbally annotated.

File Name: Supplementary Movie 12
Description: Polarized ARP2/3 complex dynamics in VEGF-induced elongated HUVEC. Confluent HUVEC cultures expressing EGFP-p20 were applied with VEGF. 24 h after VEGF application, spinning disc microscope was used to record the EGFP-p20 dynamics. Polarized EGFP-p20 dynamics at the junction indicating JAIL formation were detected at the cell pole and lateral junctions of the VEGF induced elongated cells. The video is verbally annotated.

File Name: Supplementary Movie 13
Description: Rac activity in VEGF-induced elongated HUVECs using the *Raichu-Rac1* FRET sensor. Confluent HUVEC expressing FRET sensor Raichu-Rac1 were stimulated with VEGF. Increased and polarized Rac1 activity was detected in VEGF induced elongated cells. Arrow indicates the direction of cell movement. The video is verbally annotated.

File Name: Supplementary Movie 14
Description: Rac inhibitor EHT 1864 blocks VEGF induced cell elongation, migration and JAIL formation. Confluent HUVEC cultures were pretreated for 2 h with 10 µM of EHT 1864 and subsequently exposed to VEGF. Phase Contrast microscopy after VEGF application. The video is verbally annotated.

File Name: Supplementary Movie 15
Description: The Rac-activating Sphingosine-1-Phosphate receptor activator Sew2871 upregulates JAIL formation in HUVEC. Confluent HUVEC expressing EGFP-p20 were applied with 10µM Sew2871 followed by time lapse recordings using spinning disc microscope. The video is verbally annotated.

File Name: Supplementary Movie 16
Description: Dynamics of sprouting angiogenesis in fibrin gel analyzed by a) 4D phase contrast microscopy and **b)** EGFP-p20 expressing HUVEC in a 3D fibrin angiogenesis model was reconstructed to demonstrate lumen formation. Red arrows indicate lumen formation. The movie b refers to the Supplementary Video14. Note: not all of the cells express EGFP-p20. The video is verbally annotated.

File Name: Supplementary Movie 17
Description: JAIL dynamics in 4D sprouting angiogenesis fibrin gel assay. EGFP-p20 expressed HUVEC were used in a 3D fibrin angiogenesis assay. The movie was only from one Z plane in a lumen forming vessel which was already showed in Supplementary Movie 16b. Large JAIL at the leading front and small JAIL at the lateral junctions were observed in sprouting ECs. ARP2/3 complex inhibitor CK666 inhibited JAIL formation and cell migration. The video is verbally annotated.

File Name: Supplementary Movie 18
Description: ARP2/3 complex activity during cell division in lumen forming vessels in fibrin gel assay by 4D time lapse recordings. Left panel: EGFP-p20 expressing HUVEC in a 3D fibrin angiogenesis model was reconstructed to demonstrate lumen formation. Note: not all of the cells express EGFP-p20. Right panel: EGFP-p20 dynamics during cell division in lumen forming vessel. The video is verbally annotated.
